# The Osmotin-Like Protein Gene *PdOLP1* Is Involved in Secondary Cell Wall Biosynthesis during Wood Formation in Poplar

**DOI:** 10.3390/ijms21113993

**Published:** 2020-06-02

**Authors:** Shaofeng Li, Yaoxiang Zhang, Xuebing Xin, Changjun Ding, Fuling Lv, Wenjuan Mo, Yongxiu Xia, Shaoli Wang, Jingyan Cai, Lifang Sun, Manyi Du, Chenxi Dong, Xu Gao, Xinlu Dai, Jianhui Zhang, Jinshuang Sun

**Affiliations:** 1 Experimental Center of Forestry in North China, Chinese Academy of Forestry, Beijing 100023, China; Lisf@caf.ac.cn (S.L.); zhangyx@caf.ac.cn (Y.Z.); xinxb@caf.ac.cn (X.X.); wboffice@caf.ac.cn (F.L.); wm296@cornell.edu (W.M.); yxxia@caf.ac.cn (Y.X.); wshaoli@iccas.ac.cn (S.W.); cjingyan@caf.ac.cn (J.C.); sunlf@caf.ac.cn (L.S.); dumy@caf.ac.cn (M.D.); dongcx@caf.ac.cn (C.D.); gaoxu@caf.ac.cn (X.G.); daixl@caf.ac.cn (X.D.); 2 State Key Laboratory of Tree Genetics and Breeding, Research Institute of Forestry, Chinese Academy of Forestry, Key Laboratory of Tree Breeding and Cultivation, State Forestry Administration, Beijing 100091, China; changjunding@caf.ac.cn; 3 Department of Pharmaceutical Science, Biomanufacturing Research Institute and Technology Enterprise, North Carolina Central University, Durham, NC 27707, USA

**Keywords:** functional mechanisms, negative regulator, *PdOLP1*, *Populus deltoides*, secondary wall biosynthesis

## Abstract

Osmotin-like proteins (OLPs) mediate defenses against abiotic and biotic stresses and fungal pathogens in plants. However, no OLPs have been functionally elucidated in poplar. Here, we report an osmotin-like protein designated *PdOLP1* from *Populus deltoides* (Marsh.). Expression analysis showed that *PdOLP1* transcripts were mainly present in immature xylem and immature phloem during vascular tissue development in *P. deltoides*. We conducted phenotypic, anatomical, and molecular analyses of *PdOLP1*-overexpressing lines and the *PdOLP1*-downregulated hybrid poplar 84K (*Populus alba* × *Populus glandulosa*) (Hybrid poplar 84K PagOLP1, PagOLP2, PagOLP3 and PagOLP4 are highly homologous to *PdOLP1*, and are downregulated in *PdOLP1*-downregulated hybrid poplar 84K). The overexpression of *PdOLP1* led to a reduction in the radial width and cell layer number in the xylem and phloem zones, in expression of genes involved in lignin biosynthesis, and in the fibers and vessels of xylem cell walls in the overexpressing lines. Additionally, the xylem vessels and fibers of *PdOLP1*-downregulated poplar exhibited increased secondary cell wall thickness. Elevated expression of secondary wall biosynthetic genes was accompanied by increases in lignin content, dry weight biomass, and carbon storage in *PdOLP1*-downregulated lines. A *PdOLP1* coexpression network was constructed and showed that *PdOLP1* was coexpressed with a large number of genes involved in secondary cell wall biosynthesis and wood development in poplar. Moreover, based on transcriptional activation assays, PtobZIP5 and PtobHLH7 activated the *PdOLP1* promoter, whereas PtoBLH8 and PtoWRKY40 repressed it. A yeast one-hybrid (Y1H) assay confirmed interaction of PtoBLH8, PtoMYB3, and PtoWRKY40 with the *PdOLP1* promoter in vivo. Together, our results suggest that *PdOLP1* is a negative regulator of secondary wall biosynthesis and may be valuable for manipulating secondary cell wall deposition to improve carbon fixation efficiency in tree species.

## 1. Introduction

Wood provides abundant biomass and important biomaterials for renewable sources and industrial products around the world. Wood formation includes multiple developmental stages, such as vascular cambium differentiation, vascular tissue division, cell expansion, cell wall thickening, and programmed cell death [1,2]. Cloning and transformation of genes related to wood development in tree species are important for cultivating new varieties of trees with fine quality and high yield. However, the functions of a large number of genes related to wood traits have not been discovered and the molecular mechanisms of secondary wall thickening and secondary xylem formation in forest trees remain largely unknown [3,4,5]. Hence, elucidation of the mechanisms underlying gene regulation in xylem formation and secondary cell wall synthesis during wood development would be instrumental in providing the molecular basis and technical approaches for genetic improvement of the wood properties of forest trees.

Osmotin-like proteins (OLPs) comprise a group of 24–26-kD proteins belonging to the pathogenesis-related protein 5 (PR-5) family that were originally discovered in tobacco cells (*Nicotiana tabacum* L. var Wisconsin 38) under osmotic stress conditions [6]. OLPs play important roles in growth and development. For example, higher accumulation of the Petunia (*Petunia hybrida*) osmotin-like protein PhOSM is found in the root, with lower levels detected in the leaf and flower, showing that *PhOSM* plays vital roles in normal development [7]. In addition, the rice osmotin protein gene *OsOSM1* is mainly expressed in the leaf sheath at the booting stage and is associated with development [8]. Hybrid poplar (*Populus deltoides* × *P*. *euramericana* cv. ‘Nanlin895′) *PeTLP* with high homology to *OLP* genes has been isolated. Accumulation of *PeTLP* transcripts in stems indicates that *PeTLP* is associated with wood formation [9]. Involved in stem vascular tissue development, hybrid poplar (*Populus trichocarpa* × *P. deltoides*) *TLP1*, which is homologous to *OLPs*, is mainly expressed in vascular tissues of stems, petioles, and midveins [10]. Osmotins are osmoticum-induced proteins that can accumulate in large amounts in the cytoplasmic vesicles or vacuoles of plant cells. OLPs are produced in plants under different abiotic and biotic stresses [11,12]. For instance, OLP-transgenic tomato plants under saline stress exhibit increased secondary cell wall thicknesses of fibers and vessels, which act as an important mechanism for improving the resistance of plants to water stress [13]. Abdin et al. (2011) [14] showed that osmotin proteins do not contain a DNA-binding motif and may act as modulators of metabolic signaling or transcription factors in response to biotic and abiotic stresses. In addition, osmotin proteins may have a unique contribution to pathogenesis. Indeed, osmotins exhibit antifungal activity by inhibiting spore germination and lysis, hyphal growth, and spore viability [15].

Recent studies in woody plants (e.g., poplar and eucalyptus) as well as *Arabidopsis* have demonstrated that secondary wall formation is mediated by a complex transcriptional network. In this hierarchical network of transcription factors, a group of wood-associated NAC transcription factors (WNDs), such as PtrWND2B and *Eucalyptus gunnii* EgWND1, have been identified as functional orthologs of *Arabidopsis* NACs including SND1, NST1/NST2, and VND6/VND7 and have been identified as master switches that are able to activate the promoter activities of secondary wall biosynthetic genes [16,17,18]. Several lignin biosynthetic genes in transgenic plants have been assessed in investigating the function of wood formation-related genes involved in secondary cell wall formation. Reduced transcript levels for the *PtoCCR2*, *PtoCAD1*, and *PtoC4H2* genes in *Populus trichocarpa* PtrWRKY19-overexpressing poplar plants were observed, suggesting that PtrWRKY19 negatively regulates the expression of secondary wall biosynthesis genes in poplar [19]. Furthermore, lignin biosynthetic genes, including *COMT2*, *CCOAOMT1*, *C3H*, *4CL5*, *CCR2*, *PAL4*, *HCT1*, and *CAD1* were upregulated in *P. tomentosa* (Carr.) *PtoMYB92*-overexpressing transgenic plants, suggesting that PtoMYB92 positively modulates secondary wall development in poplar [20]. *Arabidopsis thaliana* TALE homeodomain transcription factor KNOTTED ARABIDOPSIS THALIANA7 (KNAT7) interacts with a BELL1-LIKE HOMEODOMAIN protein BLH6, and this interaction may influence secondary cell wall development [21]. Sun et al., (2017) [22] showed that miR319 and its target TCP4 can activate secondary cell wall biosynthesis in *Arabidopsis*. Moreover, TCP4 possibly targets the promoter of VND7 to regulate its expression and is involved in xylem vessel differentiation. Overexpression of *SbMyb60* has been shown to promote monolignol biosynthesis and leads to induced lignin deposition and changed cell wall composition in transgenic *Sorghum bicolor* plants [23]. Additionally, Li et al. (2019) [24] showed that *Populus deltoides* (Marsh.) proline-rich protein gene *PdPRP* might positively regulate secondary cell wall formation by promoting secondary wall thickening and expansion in both poplars and *Arabidopsis*. These studies have largely improved our knowledge of the complicated regulatory mechanisms of secondary cell wall formation in poplars and other woody plants.

*P. deltoides* (Marsh.), introduced from North America into China in 1972, is a black poplar species of the *Aigeiros* section in the genus *Populus*. Compared with other poplar tree species, *P. deltoides* is resistant to stress and disease; it is fast-growing and has fine quality and good environmental adaptability. Thus, *P. deltoides* is widely used worldwide as a major species for poplar gene function studies and transgenic breeding. However, compared with knowledge regarding the biological function of OLPs in *Arabidopsis*, *Glycine max,* and *Nicotiana tabacum*, the molecular mechanism of these proteins in poplar remains largely unknown.

In this study, we isolated and characterized the molecular functions of a *P. deltoides* osmotin-like protein gene, *PdOLP1*, in secondary cell wall formation. We altered *PdOLP1* transcript levels by overexpression and suppression in the transgenic hybrid poplar 84K (*Populus alba* × *Populus glandulosa*) and analyzed its effect on growth and cell wall thickening. The *PdOLP1* coexpression network indicated that genes related to secondary cell wall biosynthesis and wood development are affected by *PdOLP1*. Moreover, transcriptional activation assays showed that several transcription factors involved in wood formation activate or repress the promoter of *PdOLP1*. Our results reveal that modifying the *PdOLP1* gene may enable cultivation of transgenic poplar lines with improved carbon sequestration and valuable properties for wood processing.

## 2. Results

### 2.1. Isolation and Characterization of PdOLP1

**Figure 1:**
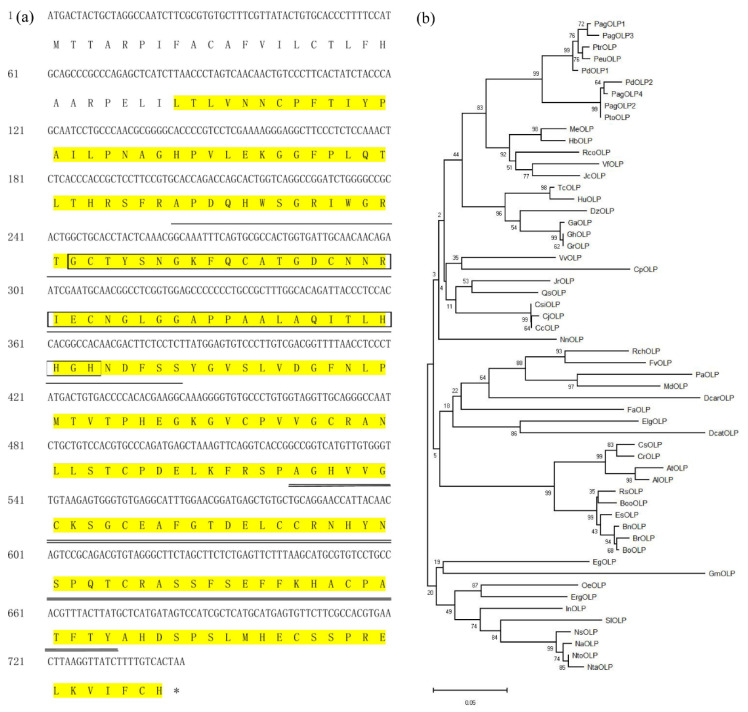
Characterization of the *PdOLP1* protein. (**a**) Amino acid sequence of the coding region of PdOPL. The sequence highlighted in yellow indicates the THN domain. The AWS domain is boxed. The VWC domain is underlined with a thin black line. FU domain is underlined with double black line. SMART (http://smart.embl-heidelberg.de/) was used to analyze the PdOPL1 protein sequence. (**b**) A phylogenetic tree of the OPL proteins from 52 plants was constructed based on the neighbor-joining method, including PdOPL1 (GenBank:MK052942), PdOLP2: (Podel.01G248000.1), PagOLP1: (Pop_G09G014129.T1), PagOLP2:.(Pop_A01G056866.T1), PagOLP3: (Pop_A09G059600.T1), PagOLP4: (Pop_G01G020795.T1), PtrOLP (GenBank:XP_024463852.1), PeuOLP (GenBank:XP_011042680.1), PtoOLP (GenBank:APA20308.1), MeOLP (GenBank:XP_021626825.1), HbOLP (GenBank:XP_021636655.1), RcOLP (GenBank:XP_002531364.1), DzOLP (GenBank:XP_022756598.1), CjOLP (GenBank:BAI63297.1), VfOLP (GenBank:ARV78462.1), TcOLP (GenBank:XP_007016468.1), CsiOLP (GenBank:XP_006488293.1), CcOLP (GenBank:XP_006424795.1), VvOLP (GenBank:XP_002281193.1), HuOLP (GenBank:XP_021278215.1), JrOLP (GenBank:XP_018823302.1), QsOLP (GenBank:XP_023907784.1), GhOLP (GenBank:XP_016689054.1), GrOLP (GenBank:XP_012446484.1), GaOLP (GenBank:XP_017650093.1), JcOLP (GenBank:XP_012065012.1), EsOLP (GenBank:XP_006409911.1), RsOLP (GenBank:XP_018463081.1), BnOLP (GenBank:XP_013676421.1), BrOLP (GenBank:XP_009140942.1), BooOLP (GenBank:XP_013632142.1), BoOLP (GenBank:AAO12209.1), RcOLP (GenBank:XP_024159050.1), OeOLP (GenBank:XP_022881590.1), FaOLP (GenBank:ABB86299.1), CsOLP (GenBank:XP_010470049.1), ErgOLP (GenBank:XP_012827362.1), FvOLP (GenBank:XP_004294572.1), InOLP (GenBank:XP_019199014.1), NtOLP (GenBank:XM_018769102.1), NnOLP (GenBank:XP_010259948.1), ElgOLP (GenBank:XP_010918389.1), EgOLP (GenBank:XP_010033142.1), NtOLP (GenBank: NP_001312698.1), PaOLP (GenBank:XP_021820385.1), AtOLP (GenBank:NP_001324474.1), AlOLP (GenBank:XP_020885214.1), DcarOLP (GenBank:XP_017251860.1), NsOLP (GenBank:XP_009800616.1), CpOLP (GenBank:XP_021887635.1), GmOLP (GenBank:XP_003538000.1), CrOLP (GenBank:XP_006294865.1), NaOLP (GenBank:XP_019237711.1), MdOLP (GenBank:XP_008357503.1). DcatOLP (GenBank:XP_020683326.1), SlOLP (GenBank:NP_001234714.1). The 51 OPL amino acid sequences are list in Table S5. Bar = 0.05 substitutions per site.

We isolated a 744 bp cDNA sequence from the immature xylem of 15-year-old *P. deltoides* (Figure 1a). The sequence has 97.6% homology with the open reading frame (ORF) of *Populus trichocarpa PtOLP*, and we named it *PdOLP1*. Another gene of *P. deltoides* (Podel.01G248000.1) has high protein similarity (93.2%) with PdOLP1, and this homologous gene was named *PdOLP2*. PagOLP1 (a gene of hybrid poplar 84K (Pop_G09G014129.T1)) exhibits high sequence similarity (97.2%) to PdOLP1. PagOLP2 (a gene of hybrid poplar 84K (Pop_A01G056866.T1)) has high sequence similarity (98.4%) to PdOLP2. PagOLP3 (a gene of hybrid poplar 84K (Pop_A09G059600.T1)) exhibits high sequence similarity (96.9%) to PdOLP1. PagOLP4 (a gene of hybrid poplar 84K (Pop_G01G020795.T1)) has high sequence similarity (89.0%) to *PdOLP1* [25]. 

Furthermore, sequence analysis showed that the cDNA sequence of *PdOLP1* is 74–97.6% identical to the cDNA sequences of OLPs from 51 other plant species. *PdOLP1* encodes a protein of 247 amino acids (Figure 1a) with a predicted molecular mass of 26.85 kD and a calculated isoelectric point (pI) of 7.86.

Comparison of *PdOLP1* with 51 OLPs from other species revealed high homology between PdOLP1and PeuOLP (97% similarity) in *P. euphratica*, PtoOLP (88% similarity) in *P. tomentosa*, HbOLP (86% similarity) in *Hevea brasiliensis*, MeOLP (79% similarity) in *Manihot esculenta*, EgOLP (79% similarity) in *Eucalyptus grandis*, and AtOLP (72% similarity) in *Arabidopsis thaliana* (Figure 1b, Table S6).

The *PdOLP1* protein sequence contains some typical protein motifs, including a THN domain, a VWC domain, and an AWS domain (Figure 1a, Figure S2). A THN domain that belongs to the thaumatin family and is also referred to as pathogenesis-related group 5 (PR5) exists between residues 28 and 274; it is related to plant pathogenesis and possesses antifungal activity. The VWC domain between residues 68 and 130 is involved in transcription, signal transduction, ribosomal and membrane transport and the proteasome. The AWS domain is situated between residues 82 and 123, with a role in gene regulation by methylation of lysine residues in histones and other proteins. Furin-like repeats of the FU domain, involved in the mechanism of signal transduction by receptor tyrosine kinases, may be present between residues 175 and 224. These analyses indicate that the *PdOLP1* protein may play an important role in signal transduction, transcription, and methylation, among other processes.

### 2.2. Expression Pattern and Subcellular Localization of PdOLP1

**Figure 2:**
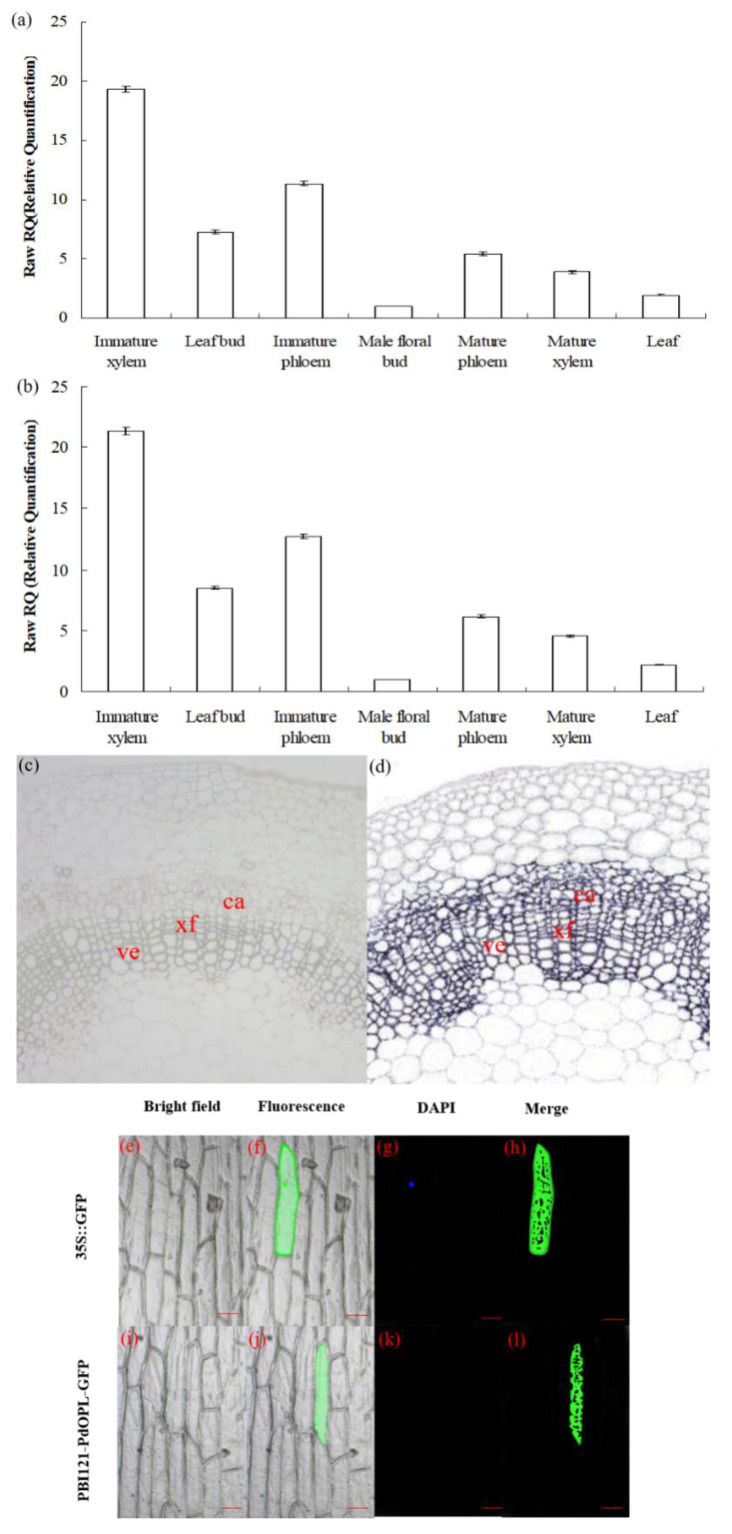
Expression pattern and subcellular localization of the *PdOLP1* protein. (**a**,**b**) *PdOLP1* expression in various tissues was analyzed by quantitative real-time PCR (qRT-PCR). Expression was normalized to α-tubulin (*TUA1*) (**a**) and ubiquitin (*UBQ1*) reference genes (**b**). Error bars: ± standard error (SE) of three replicates. (**c**,**d**) In situ localization of *PdOLP1* in *P. deltoides* stems. Sections of the stems were hybridized with digoxigenin-labeled antisense (**c**) or sense (**d**) probes. The hybridization signals are shown in purple. ve, vessel; xf, xylary fiber; ca, cambium. Scale bars = 25 µm. (**e**–**l**) Subcellular localization of PdOLP1. The green fluorescent signal of PBI121-*PdOLP1*-*GFP* was detected within the cytoplasm of onion epidermal cells (**j**,**l**), and green fluorescent protein (GFP) signal alone was localized in the cytoplasm, nucleus (4′,6-diamidino-2-phenylindole (DAPI) stained) and plasma membrane (**f**,**h**). Scale bars = 100 µm.

Quantitative real-time PCR (qRT-PCR) analysis revealed that *PdOLP1* was differentially expressed at detectable levels in all tissues as tested. The relative expression levels of *PdOLP1* in immature xylem and immature phloem of 15-year-old *P. deltoides* were higher than in any other examined tissues, whereas expression in the floral bud was the lowest (Figure 2a). The expression levels of *PdOLP1* in different tissues were almost the same when using either *TUA1* (Figure 2a) or *UBQ1* (Figure 2b) as internal controls. In situ hybridization analysis of *P. deltoides* stem segments indicated the *PdOLP1* gene to be specifically expressed in xylary fibers and vessels, with lower expression levels found in male floral buds (Figure 2c). There was no detectable hybridization signal in the control sections hybridized with *PdOLP1* sense probes (Figure 2d). These results suggest that *PdOLP1* is expressed predominantly in the immature xylem and immature phloem tissues undergoing secondary cell wall thickening. The *PdOLP1* coding sequence was fused in-frame to the 5′ end of green fluorescent protein (GFP) (Figure S1); subcellular localization of PBI121-PdOLP1-GFP showed green fluorescence of the recombinant protein in the cytoplasm of onion epidermal cells (Figure 2j,l). In contrast, using the construct with GFP alone used as a control, green fluorescence was observed throughout the cells, including the cytoplasm, plasma membrane, and nucleus (4′,6-diamidino-2-phenylindole (DAPI) stained) (Figure 2f,h). These results suggest that PdOPL is located in the cytoplasmic compartment.

### 2.3. Verification of PdOLP1 Overexpression and Downregulation in Transgenic Poplar Plants

**Figure 3:**
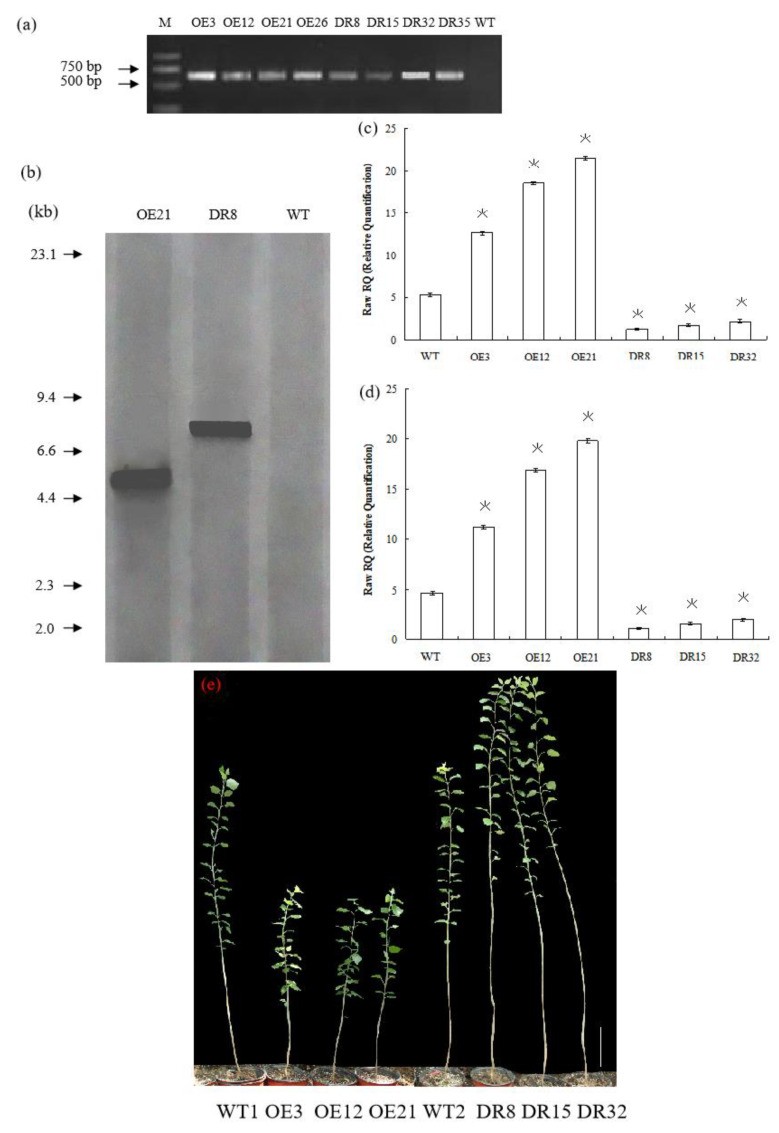
*PdOLP1* overexpression restricted development, and *PdOLP1* downregulation promoted growth. (**a**) Transgenic poplar plants were identified by amplifying the *NptII*-sensitive marker (Table S3). M, DNA molecular weight marker; O3, O12, and O21, *PdOLP1*-overexpressing poplar plants; DR8, DR15 and DR32, *PdOLP1*-downregulated poplar plants. (**b**) Southern blot analysis of PCR identified transgenic poplar plants. Genomic DNA of the plants digested with *XbaI* was hybridized with the *NptII* probe. OE21, *PdOLP1*-OE hybrid transgenic poplar line; DR8, *PdOLP1*-DR poplar line; WT, wildtype. (**c**,**d**) Confirmation of *PdOLP1* expression levels in the transgenic plants by qRT-PCR. *TUA1* (**c**) and *UBQ1* (**d**) were used as reference genes. Error bars represent the standard deviation from the mean. (**e**) Growth comparison of 150-day-old WT1 poplar and the *PdOLP1*-OE lines OE3, OE12 and OE21. Growth comparison of 150-day-old WT2 poplar and the *PdOLP1*-DR lines DR8, DR15 and DR32 (right). The scale bars correspond to 21.71 cm/in (**e**).

To investigate the biological function of *PdOLP1*, we overexpressed it under the control of the proNAC068 promoter in hybrid poplar (*P. alba* × *P. glandulosa*) of clone 84K. A total of 28 independent overexpression lines (pBI121*-ProNAC068-sense PdOLP1*) and 35 suppression lines (pBI121*-ProNAC068-antisense PdOLP1*) were obtained and examined by PCR (Figure 3a). At least one copy of the *PdOLP1* gene was detected in three of the overexpression (OE3, OE12, OE21) and three of the knockdown (DR8, DR15, DR32) lines (Figure 3b) by southern hybridization, and no hybridization signals were detected in wildtype plants. This indicates that the constructs containing *PdOLP1* had integrated into the genomes of the transformed poplar plants. qRT-PCR was used to further verify transgene expression in *PdOLP1*-overexpressing (OE) lines and *PdOLP1*-downregulated (DR) lines, revealing significant upregulation of *PdOLP1* by 137.67%, 247.53%, and 303.65% in the *PdOLP1*-OE poplar lines OE3, OE12, and OE21, respectively, compared with non-transgenic (WT) poplar plants (*p* = 0.002, Figure 3c,d). *PdOLP1* expression in the three independent *PdOLP1*-DR lines DR8, DR15 and DR32 was obviously reduced by 77.00%, 67.12%, and 59.64%, respectively, compared with the WT plants (*p* = 0.008, Figure 3c,d). The expression patterns found using *TUA1* (Figure 3c) as an internal control were the same as those using *UBQ1* (Figure 3d). These six transgenic poplar lines (OE3, OE12, OE21, DR8, DR15, and DR32) with higher or lower level expression of *PdOLP1* were selected for further functional characterization.

### 2.4. Overexpression of PdOLP1 Delayed Primary Growth and Repressed Secondary Wall Biosynthesis in Poplars

**Figure 4:**
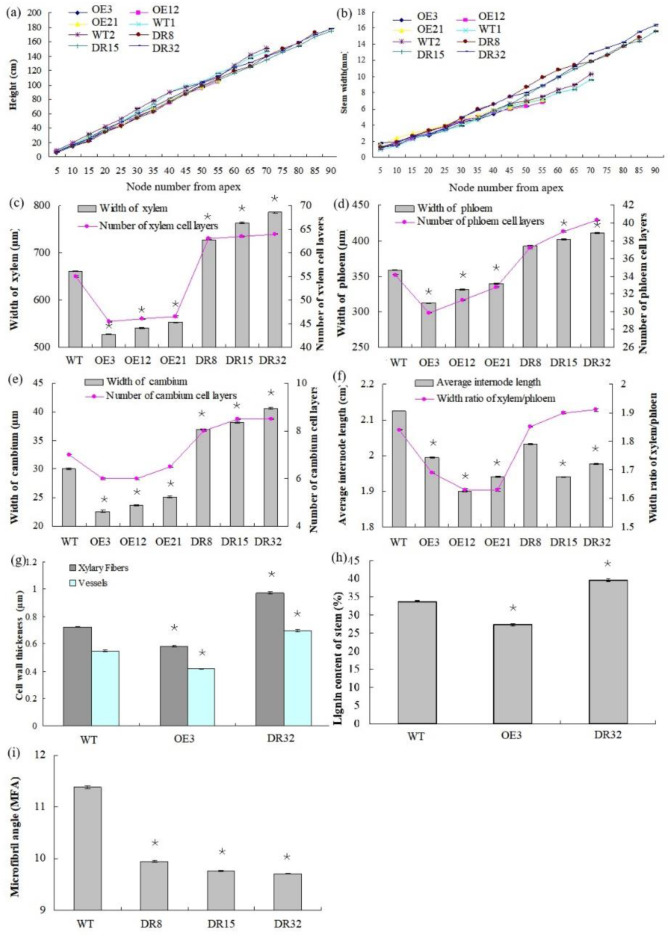
Anatomical features of the stem cross-sections, growth, and wood property indexes of *PdOLP1* transgenic plants. (**a**,**b**) *PdOLP1*-OE plants showed smaller shoots and a decreased stem diameter compared to WT plants. Plant height (**a**) and stem width (**b**) were measured at every fifth node from the apex. (**c**) Xylem widths (left panel) and numbers of xylem cell layers (right panel) in transgenic or WT plants. (**d**) Phloem widths (left panel) and numbers of phloem cell layers (right panel) in transgenic or WT plants. (**e**) Cambium widths (left panel) and numbers of cambium cell layers (right panel) in transgenic or WT plants. (**f**) The average internode lengths (left panel) and the xylem: phloem width ratios (right panel) in transgenic or WT lines. (**g**) Thicknesses of vessels and fibers in the stems of transgenic or WT plants. The values are the mean ± standard error (SE) of 20 cells. (**h**) Lignin content of transgenic or WT plants. (**i**) Analysis of the microfibril angle (MFA) in *PdOLP1* transgenic poplar plants. The *PdOLP1*-DR poplar lines had lower MFAs than the control line. Significant differences between the transgenic plants and WT plants are marked with asterisks (* *p* < 0.05). WT: wildtype poplar; WT1: control for *PdOLP1*-OE plant; WT2: control for the *PdOLP1*-DR plant; OE3, OE12 and OE21: *PdOLP1*-OE poplar lines; DR8, DR15 and DR32: *PdOLP1*-DR plants.

**Figure 5:**
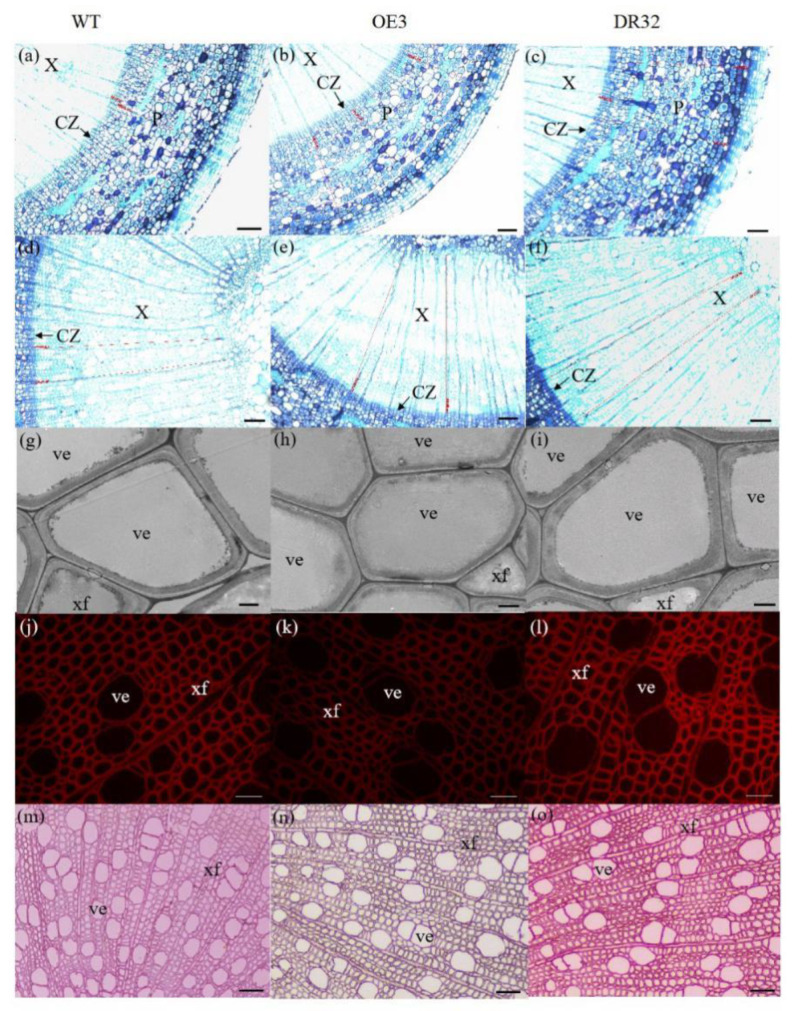
Anatomical transverse sections of *PdOLP1* transgenic poplar. The 15th internodes of 1.5-year-old plants were used to observe the anatomical characteristics of the stem cross-sections. (**a**–**f**) Toluidine blue staining of stem sections of a WT plant (**a**,**d**), *PdOLP1*-OE-transgenic plant (**b**,**e**) and *PdOLP1*-DR-transgenic plant (**c**,**f**). Scale bars = 100 µm. (**g**–**i**) Scanning electron microscopy of sections analyzed in WT (**g**), OE (**h**) and DR (**i**) plants. Scale bars = 5 µm. (**j**–**l**) Lignin autofluorescence was observed in WT (**j**), OE (**k**) and DR (**l**), Scale bars = 400 µm. (**m**–**o**) Phloroglucinol-HCl staining of WT (**m**), OE (**n**) and DR (**o**) plants. Scale bars = 400 µm. C, cortex cells; CZ, cambial zone; Pi, pith; P, secondary phloem; ve, vessel; X, secondary xylem; xf, xylem fiber.

After growth for 1.5 years in a greenhouse with a 16/8 h light/dark cycle, *PdOLP1*-OE transgenic lines OE3, OE12 and OE21 showed 28.14%–34.47% reduction in plant height and a reduced diameter of 28.27–32.97% compared to WT plants (Figure 4a,b), respectively. Overexpression of *PdOLP1* in hybrid poplar resulted in decreased radial width and number of cell layers of the xylem, cambium and phloem zones of the 15th internode compared with controls (Figure 4c–e, Figure 5a,b,d,e, Table S7). Reductions were also detected in the number of internodes, width ratio of xylem/phloem and average internode length in *PdOLP1*-OE plants (Figure 4f). Moreover, the cell walls of xylem fibers and vessels in the three *PdOLP1*-OE lines were significantly thinner than those in WT plants (*p* = 0.013, Figure 5g,h). To determine lignin modifications, we measured the Klason lignin content in the stems of WT and transgenic plants. The three tested *PdOLP1*-OE lines contained 18.99% lower lignin contents on average than WT plants (*p* = 0.003, Figure 4h). Weaker lignin autofluorescence was also observed in *PdOLP1*-OE OE3 plants compared to WT plants (Figure 5j,k). Phloroglucinol-HCl staining was employed to visualize lignin deposition and distribution in cell walls, and the 15th internodes of OE3 plant stem sections showed weaker staining in both the xylem vessels and interfascicular fibers (Figure 5m,n). These results demonstrate that upregulation of *PdOLP1* weakened lignin deposition in transgenic plants and that *PdOLP1* may negatively regulate secondary cell wall thickening of fibers and vessels in poplar stems.

### 2.5. Downregulation of PdOLP1 Promotes Vigorous Growth and Causes Ectopic Thickening of the Secondary Wall in Poplar

1.5-year-old *PdOLP1*-downregulated poplar lines displayed evident alterations in primary growth; plant heights were increased at least 16.10%, and stem widths were 56.88% thicker than those of WT plants (Figure 4a,b). The *PdOLP1*-downregulated lines individually produced more secondary xylem and secondary phloem than did the WT plants, with a mean increase in xylem radial width of 14.75% and an increase of 15.45% in xylem cell layers (Figure 4c, Figure 5a,c,d,f, Table S7).

Based on quantitative analysis, the cell wall thicknesses of the xylem fibers and vessels of the three *PdOLP1*-DR plants were significantly thicker than those of WT plants (*p* = 0.009, Figure 5g,i). Thus, lignin accumulation was significantly increased by 17.51% in stems of *PdOLP1*-DR poplars compared to WT plants (*p* = 0.005, Figure 4h). Confocal microscopy of lignin autofluorescence revealed lignified secondary wall thickening in WT stem cross-sections, but with higher signals in *PdOLP1*-downregulated DR8 plants (Figure 5j,l). Phloroglucinol-HCl staining of lignin in stem sections showed an intense red stain of the secondary cell walls in DR8 plants (Figure 5m,o), but less intense staining was detected in WT plants (Figure 5m). Therefore, downregulation of *PdOLP1* in poplar causes high levels of lignification in stem xylem cells. 

### 2.6. Wood Index, Total Biomass, and Carbon Storage Is Obviously Changed in PdOLP1-DR Poplars

The microfibril angle (MFA) values of 1.5-year-old *PdOLP1*-transgenic plants were measured, and a reduction of 12.69–14.86% was found compared to WT plants, with statistically significant differences (*p* = 0.005, Figure 4i). The *PdOLP1*-DR transgenic plants also had a significantly higher wood density, with up to a 20.39% increase compared to WT plants. Therefore, it is possible that *PdOLP1* may play an important role in improving wood mechanical properties in poplar. Obvious differences in shoot and root biomass between transgenic and nontransgenic plants were also found when dry weight was analyzed. *PdOLP1*-DR plants had a 30.05% higher dry weight biomass than WT plants (*p* = 0.008), whereas the dry weight biomass in *PdOLP1*-OE plants was decreased by 22.56% compared to WT (*p* = 0.006, Table S8). Biotic sequestration is an effective way to weaken the influence of continuously rising atmospheric CO_2_ concentrations through increased carbon sequestration by forest trees [26]. Indeed, the carbon stock was significantly enhanced by 31.63% in the three tested *PdOLP1*-DR poplar lines (*p* = 0.005, Table S8), suggesting improved carbon storage.

### 2.7. Overexpression or Downregulation of PdOLP1 Affects Expression of Cell Wall Biosynthetic Genes in Transgenic Poplars

**Figure 6:**
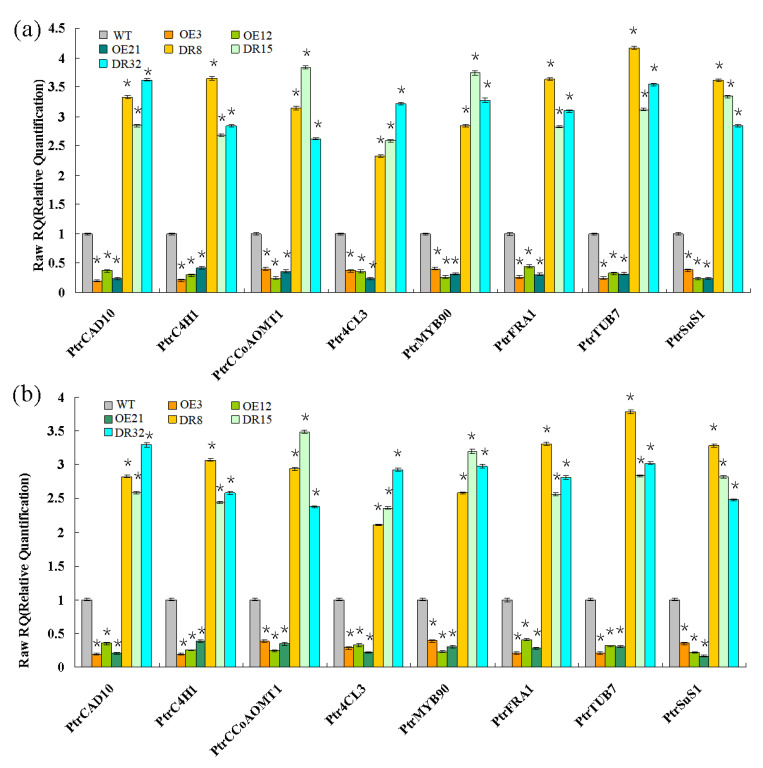
Wood formation-related genes analyzed by qRT-PCR. Eight genes involved in secondary wall biosynthesis were evaluated: *Ptr4CL3*, *PtrCAD10*, *PtrC4H1,* and *PtrCCoAOMT1*, Ptr*MYB90, PtrFRA1*, *PtrSuS1,* and *PtrTUB7*. *TUA1* (**a**) and *UBQ1* (**b**) were used as internal control genes, and transcript levels of genes in WT plants were set to 1. The data are the mean ± SE from three biological replicates. Asterisks indicate significant differences between transgenic and WT plants (* *p* < 0.05).

Expression of secondary wall biosynthetic genes was investigated in 1.5-year-old *PdOLP1* transgenic poplar. Two internal control genes and eight genes associated with the phenylpropanoid pathway and microfibril angle formation were selected to confirm primer amplification efficiency and specificity (Table S4, Figures S3 and S4). The results from qRT-PCR analysis revealed that the transcript levels of *Ptr4CL3*, *PtrCAD10*, *PtrC4H1*, *PtrCCoAOMT1,* and PtrMYB90 were significantly repressed in the *PdOLP1*-OE poplar plants (Figure 6). In addition, the expression of microfibril angle-related genes *PtrSuS1*, *PtrTUB7,* and *PtrFRA1* was obviously downregulated in *PdOLP1*-OE poplar compared with WT plants (Figure 6). However, downregulation of *PdOLP1* in poplar resulted in upregulation of the eight genes examined. Compared with WT plants, in the *PdOLP1*-DR lines, the expression level of *Ptr4CL3* increased by 132.30%–221.80% (*p* = 0.004), and that of *PtrCCoAOMT1* increased by 152.30–274.10% (*p* = 0.006); in the *PdOLP1*-OE lines, *PtrSuS1* expression decreased by 61.2–75.10% (*p* = 0.003) and *PtrCAD10* decreased by 62.6–79.80% (*p* = 0.002). These results of these experiments indicate that *PdOLP1* may function as a negative regulator of secondary wall biosynthesis in poplar.

### 2.8. Coexpression Network of PdOLP1 in Poplars

**Figure 7:**
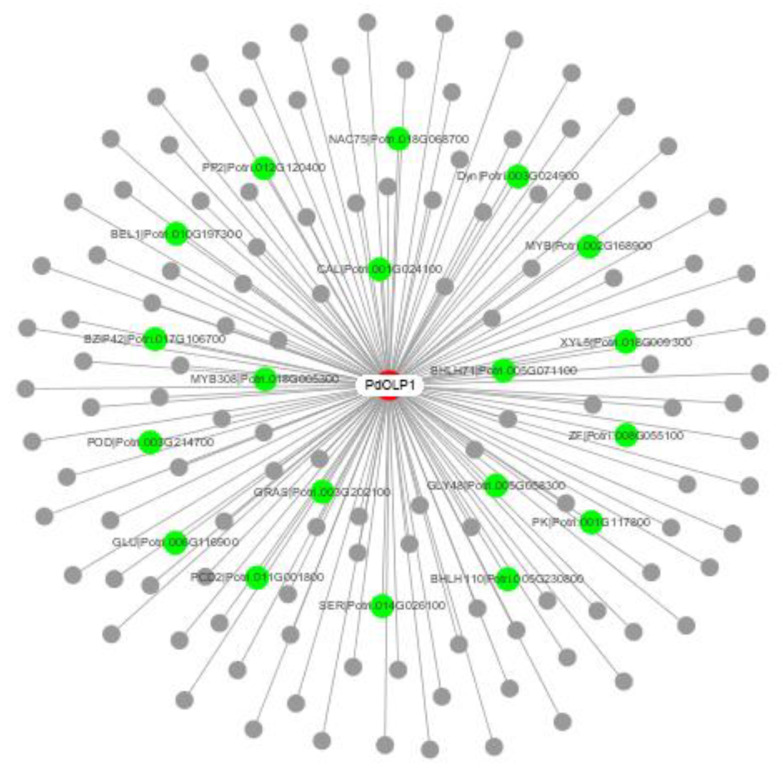
*PdOLP1* coexpression network. Among the 140 genes coexpressed with *PdOLP1*, 19 (green nodes) are involved in wood formation based on previous research. The white node is *PdOLP1;* gray nodes are other coexpressed genes.

To elucidate the molecular mechanism of *PdOLP1* in poplar growth and development, a *PdOLP1* coexpression network was constructed (Figure 7 and Table 1). A total of 140 genes were identified in the coexpression network. Among these, 19 genes (15.70%) are associated with wood formation. For example, NACs are involved in the regulation of secondary cell wall biosynthesis and zinc finger protein (ZFs)-induced secondary cell wall thickening in the stem, bZIP can bind to the regulatory element that controls xylem-specific gene expression, glycosyl transferases participate in cell wall biosynthesis, and glucomannan-synthases are believed to have a role in hemicellulose biosynthesis. bHLH transcription factor regulates early xylem development, and MYBs play a vital role in regulating secondary wall formation; several bHLH and MYB transcription factor members including bHLH71, bHLH110, and MYB308 were coexpressed with *PdOLP1*. Furthermore, some genes related to protein kinase family proteins such as leucine-rich-repeat receptor-like kinase, which is associated with fiber development and secondary cell wall formation, were coexpressed with *PdOLP1*. 

**Figure 8:**
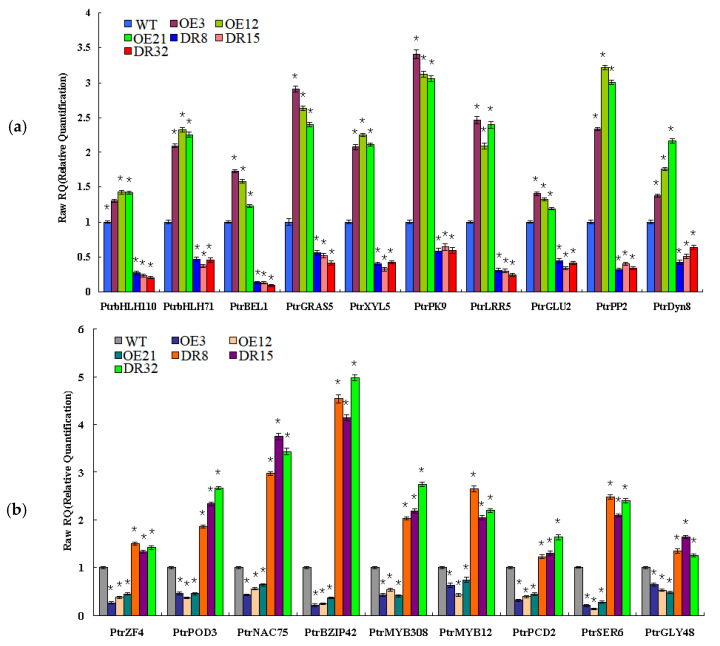
Expression patterns of secondary cell wall biosynthesis genes from the coexpression network in *PdOLP1*-OE and *PdOLP1*-DR plants and poplar protoplasts. (**a**) Expression patterns of wood formation-related genes upregulated in *PdOLP1*-OE plants and downregulated in *PdOLP1*-DR plants. (**b**) Expression patterns of secondary wall biosynthesis genes upregulated in *PdOLP1*-DR plants and downregulated in *PdOLP1*-OE plants. *UBQ1* was used as a reference gene, and the expression level of each gene in the WT background was set to 1. Asterisks indicate a significant difference between the WT and the transgenic lines (* *p* < 0.05).

To validate the network constructed by the core of *PdOLP1*, changes in the expression of the 19 genes related to wood formation in the *PdOLP1* coexpression network were compared in 3-month-old *PdOLP1*-OE, *PdOLP1*-DR and WT plants. Among the 19 genes, 10 were upregulated in *PdOLP1*-OE plants, and 9 were upregulated in *PdOLP1*-DR plants (Figure 8). Of the genes upregulated in *PdOLP1*-OE plants, 2 were upregulated by >2-fold in *PdOLP1*-OE lines, such as PtrPK9 and PtrPP2 (2.0- to 2.5-fold) (Figure 8). Of the upregulated genes in *PdOLP1*-DR plants, 3 were upregulated by >2-fold (*p* value <0.5), such as PtrNAC75 and PtrbZIP42 (2.0- to 4.0-fold) (Figure 8). The expression patterns of coexpressed genes in transgenic poplar plants suggest that *PdOLP1* cooperates with a large number of wood-formation-related genes to regulate secondary growth and development.

### 2.9. The PdOLP1 Promoter Is Activated by PtobZIP5 and PtobHLH7, and Inhibited by PtoBLH8 and PtoWRKY40

**Figure 9:**
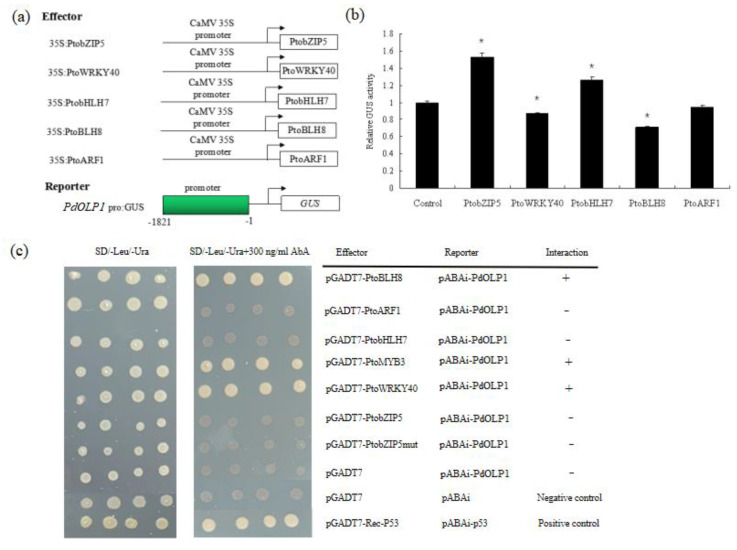
Activation of the *PdOLP1* promoter by PtobZIP5 and PtobHLH7 and inhibition by PtoBLH8 and PtoWRKY40. (**a**) Diagrams of the reporter and effector constructs used for transactivation analysis. (**b**) Transcriptional activity assays in tobacco leaves showed that PtoBLH8 and PtoWRKY40 repress *PdOLP1* promoter-driven GUS expression and that PtobZIP5 and PtobHLH7 activate GUS reporter gene expression. GUS expression in tobacco leaves not transfected with any effector was used as a control and was set to 1. Values are the mean ± standard error (SE) of three biological replicates. (**c**) Yeast one-hybrid (Y1H) assay showing the interaction between PtoBLH8, PtoMYB3, and PtoWRKY40 and the *PdOLP1* promoter. The yeast strains were grown on SD/-Leu/-Ura and SD/-Leu/-Ura +300 ng/mL Aureobasidin A (AbA) for 3 d.

Several binding motifs such as “ACACNNG”, “TGAC”, and “CANNTG” were detected in the promoter of *PdOLP1* (Table S9). This suggests that wood-associated transcription factors such as bZIP, WRKY, bHLH, BELL1-LIKE HOMEODOMAIN protein (BLH) and auxin response factor (ARF) may regulate *PdOLP1* expression through promoter binding. In this study, we employed a transient expression assay in tobacco to investigate whether the *PdOLP1* promoter can be activated or inhibited by PtobZIP5, PtoWRKY40, PtobHLH7, PtoBLH8 and PtoARF1. Subsequent assays of GUS activity in transiently transfected tobaccos demonstrated that PtobZIP5 and PtobHLH7 were able to activate expression of *PdOLP1* and that PtoBLH8 and PtoWRKY40 were able to inhibit its expression. However, there were some differences in the activation or inhibition levels. PtobZIP5 and PtobHLH7 increased *PdOLP1* activation by 53.36% and 26.63% compared to the control, respectively. PtoBLH8 and PtoWRKY40 decreased *PdOLP1* expression by 29.27% and 13.42% compared to the control, respectively (Figure 9b).

Regulation of *PdOLP1* gene expression by these transcription factors was confirmed by a Y1H assay, which detected interaction of the PtoBLH8, PtoMYB3, and PtoWRKY40 protein with the *PdOLP1* promoter in vivo (Figure 9c). The full-length PtobZIP5, PtoWRKY40, PtoMYB3, PtobHLH7, PtoBLH8, and PtoARF1 gene and the *PdOLP1* promoter fragments were cloned into pGADT7 and ABAi vectors, respectively. Although yeast strains carrying the pGADT7-PtoBLH8, pGADT7-PtoMYB3, and pGADT7-PtoWRKY40 constructs grew normally on selective medium, the empty vector control did not, indicating a direct interaction between PtoBLH8, PtoMYB3, and PtoWRKY40 protein with the *PdOLP1* promoter, respectively (Figure 9c).

## 3. Discussion

Secondary walls of woody plants display various changes in their properties and composition [27,28]. These differences are reflected in the cell wall components and thickness, cell morphology and size, cell layers and width of the xylem or phloem region. Isolating and transforming genes that regulate secondary wall thickening and development in plants can be useful in analyzing the molecular mechanism of wood-formation-associated proteins. OLPs were previously found to play vital roles in plant organ or tissue development. Pluskota et al. (2019) reported that tomato (*Solanum lycopersicum* L.) *SlNP24*, encoding an osmotin protein, was mainly localized in the micropylar region of tomato seed endosperm, and plays an important role in germinating tomato seeds [29]. Previous studies have also identified that osmotin or OLPs accumulate in response to biotic and abiotic stresses, such as drought, salt, or cold stress, and possible mechanisms underlying the defense function of OLPs against biotic and abiotic stresses were proposed [11,12]. In the present study, we demonstrate a new mechanism by which the poplar OLP gene *PdOLP1* is associated with secondary cell wall development, and the effect of *PdOLP1* overexpression or downregulation on secondary cell wall formation was investigated in poplar.

According to the study of Raghothama et al. (1997), expression of the fusion vector consisting of an osmotin promoter fused with a GUS reporter gene was increased in the stem, leaves, and flowers of transgenic tobacco, with the greatest increases in the epidermis and vascular parenchyma of the stem [30]. Chowdhury et al. (2017) detected tissue-specific expression of sesame *SindOLP* in the stems, leaves, roots, and fruit of *SindOLP*-transgenic line-7 [31]. Wang et al. (2013) isolated hybrid poplar (*Populus deltoides* × *P*. *euramericana* cv. ‘Nanlin895′) *PeTLP*, which show high homology to *OLP* genes. Increased accumulation of *PeTLP* transcripts in stems but low transcription levels detected in roots and leaves have also been reported [9]. Additionally, Dafoe et al. (2010) [32] cloned hybrid poplar (*Populus trichocarpa* × *P. deltoides*) *TLP1*, which is homologous to *OLPs*, expressed predominantly in the vascular tissues of stems, midveins and petioles and localizes in phloem parenchyma cells [10]. In our research, the highest level of *PdOLP1* expression was found in immature xylem and immature phloem (Figure 2a,b). This tissue-specific expression pattern of *PdOLP1* in *P. deltoides* is in accordance with the expression of *PeTLP* in hybrid poplar (*Populus deltoides* × *P*. *euramericana* cv. ‘Nanlin895′) [9] and *TLP1* in hybrid poplar (*Populus trichocarpa* × *P. deltoides*) [10]. Such preferential expression in immature xylem and phloem tissues indicates that *PdOLP1* is involved in secondary wall development and wood formation in poplar.

Green fluorescent protein (GFP) is used as a marker of protein localization and provides a method to study gene function [33,34]. In recent years, stable or transient GFP expression has been carried out in the model plant *Arabidopisis thaliana*, rice, poplars and other plants [35,36]. DNA particle bombardment can be used to obtain transgenic poplar [37], rice cowpea [38], peanut [39], bean [40], and soybean [41]. Kim et al. (2010) [42] found that lemon (*Citrus jambhiri* Lush.) can also be used for this. *RlemTLP* encodes a thaumatin-like protein that is homologous to osmotin. Subcellular localization analysis showed that GFP-tagged RlemTLP predominantly localized to both the periphery of the cytoplasm and the plasma membrane [42]. In our study, the recombination protein PBI121-*PdOLP1*-*GFP* localized to the cytoplasm in onion epidermal cells (Figure 2e–l). This cytoplasm localization of PBI121-*PdOLP1*-*GFP* protein coincides with the subcellular localization of the *OLP* gene in other plants, such as lemon [42].

Expression effects of target genes under the control of the *CaMV 35S* promoter is stable and continuous, with no clear differences among various organs/tissues or between different developmental stages. However, constitutive and stable transgene expression is not beneficial to the long-term growth and development of transgenic plants [43,44]. Tissue-specific or inducible promoters can be used for driving target gene expression in specific tissues or locations. NAC transcription factors act as upstream switches associated with secondary cell wall development and vascular tissue differentiation [16,17,18]. In previous studies of ProNAC068::GUS-transgenic poplar plants (the promoter of *P. deltoides* PtoNAC068 replaced the *CaMV 35S* promoter), GUS expression was mainly detected in vascular tissues, especially in xylem regions of stem cross-sections compared to the untransformed plants [45]. Therefore, the ProNAC068 promoter is tissue specific, and can be used to study the function of target genes by regulating genes specifically expressed in vascular tissues. To further analyze the role of *PdOLP1* in regulating secondary wall biosynthesis, we replaced the *CaMV 35S* promoter with the ProNAC068 promoter to drive *PdOLP1* expression specifically in vascular tissues. Compared with untransformed poplar plants, expression of *PdOLP1* was increased by 137.67–303.65% in *PdOLP1*-OE (*PdOLP1* driven by the *ProNAC068* promoter) poplar, and secondary wall deposition and expansion were detected in *PdOLP1*-OE poplar lines.

The sequence similarity of the four genes (PagOLP1, PagOLP2, PagOLP3, and PagOLP4) in hybrid poplar 84K is very high, more than 88%. Although we designed multiple pairs of primers, we unable to distinguish the expression of each gene. Therefore, we believe that the phenotype of *PdOLP1*-downregulated poplar is caused by inhibition of multiple endogenous genes of hybrid poplar 84K and does not merely involve suppression of the target gene (*PdOLP1*). Because of the high similarity of these endogenous genes, if we only inhibit the expression of one of the most homologous endogenous genes of hybrid poplar 84K, such as PagOLP1, the expression of other highly homologous endogenous genes can reverse the repressive effect of this endogenous gene (PagOLP1), and we may not observe a phenotype caused by gene inhibition. Antisense suppression can effectively inhibit the expression of several endogenous genes; thus, the function of the homologous gene cannot be complemented by other highly homologous genes. We generated *PdOLP1*-overexpressing and *PdOLP1*-downregulated hybrid lines. The phenotype of antisense inhibition is opposite to that of overexpression, which showed that the phenotype obtained by antisense inhibition was correct. Therefore, antisense inhibition was used to suppress multiple endogenous genes, resulting in a phenotype opposite to that of gene overexpression. Accordingly, the function of this gene can be confirmed from two aspects: gene overexpression and gene inhibition.

Plant cell walls have heterogeneous structures that affect xylem or phloem cell morphology and size and provide a supporting structure to promote plant growth and development [19,24]. Dafoe et al. (2010) detected expression of TLP (*OLPs* homologous gene) in sieve elements and phloem parenchyma cells by using a TLP antiserum [10]. Another study found that tomato *OLP*-transgenic plants exhibit increases in root biomass and total biomass both in the normal state and under stress conditions [43]. In our research, phloroglucinol–HCl staining and lignin autofluorescence revealed more fluorescent signals in *PdOLP1*-DR DR8 plants (Figure 5j,l,m,o), indicating that *PdOLP1* downregulation induced lignin deposition. Moreover, *PdOLP1* downregulation increased primary growth (such as stem elongation and enlargement) and dry weight biomass and facilitated secondary wall thickening and expansion in xylem zones. The radial widths and number of cell layers in the cambium, xylem and phloem tissues and the secondary cell wall thickness of vessels and fibers were significantly increased in *PdOLP1*-DR poplars (Figure 4c–e,g). This result is similar to the findings in *Betula platyphylla BplMYB46*-overexpressing birch plants, whereby the fresh weight, lignin deposition, and secondary cell wall thickness were increased in *BplMYB46*-overexpressing birch plants [46]. Together, these studies suggest that *PdOLP1* may function as a negative regulator that promotes ligninification and secondary wall deposition and therefore plays a vital role in poplar development under normal or stress conditions.

Microfibril angle (MFA) is a wood chemical parameter characteristic of strength and stiffness [47]. A lower MFA indicates higher lumber strength and rigidity [48], which is valuable for improving wood mechanical properties in forest tree breeding. The MFA in *Pinus patula* was measured and it influences the modulus of elasticity of wood [49]. Derba-Maceluch et al. (2015) downregulated hybrid poplar (*Populus tremula* × *tremuloides*) *PtxtXyn10A* and altered the cellulose MFA in lumber fibers [50]. A previous study revealed that the MFA values in transgenic hybrid poplar (*P*. *davidiana* × *P*. *bolleana*) expressing antisense *PdREM*, *PdRanBP,* or *PdCYTOB* constructs were significantly lower those in untransformed plants [28,51,52]. In this research, the stem MFAs of *PdOLP1*-DR poplars were 12.69%–14.86% lower than in non-transgenic lines (Figure 4i). Therefore, *PdOLP1* can contribute to physical mechanical strength improvement in poplar wood. Indeed, expression of several MFA-associated genes, such as *PtrSuS1* [53], *PtrFRA1* [54], *PtrTUB7* [55], and *PtrCCR7* [56], was increased in *PdOLP1*-DR plants, suggesting the functional mechanism by which the MFA was reduced in the transgenics plants.

To further elucidate the molecular mechanism of *PdOLP1* in poplar, a coexpression network mediated by *PdOLP1* was constructed (Figure 7 and Table 1). Consistent with the potential role of *PdOLP1* as a negative regulator, most positive transcriptional regulators in the previously constructed network were repressed in *PdOLP1*-OE plants. For example, Zhong et al. (2011) [18] found that *P*. *trichocarpa* PtrNAC, PtrMYB, and PtrZF transcription factors can activate expression of lignin, xylan and cellulose biosynthesis genes in poplar. In our study, four (PtrNAC75, PtrZF4, PtrMYB308, and PtrMYB12) of these positive transcription factors were repressed in *PdOLP1*-OE plants, which may indirectly suggest that lignin synthesis genes were downregulated in *PdOLP1*-OE poplar plants. bHLH transcription factors are important regulators of development in plant. Yan et al. (2013) [57] overexpressed *Sorghum bicolor SbbHLH1* in *Arabidopsis thaliana,* markedly downregulating the lignin biosynthesis genes *COMT*, *HCT*, *PAL1*, *CCR1*, and *4CL1*, and reducing the lignin content of transgenic plants. In this study, two bHLH genes, PtrbHLH110 and PtrbHLH71, were upregulated in *PdOLP1*-OE plants and may downregulate the lignin synthesis genes and decrease the lignin content. Sasaki et al. (2006) found that *P*. *alba* cell wall peroxidase-cationic (CWPO-C) is essential for the lignification of the secondary xylem [58]. We found that *PtrPOD3* was upregulated in *PdOLP1*-DR lines, indicating that peroxidase is responsible for promoting lignification of the cell wall in *PdOLP1*-DR poplar lines. Additionally, Liu et al. (2014) overexpressed *PtrBEL1* in *Arabidopsis* and revealed a decreased cell wall thickness of interficular fibers (IF) compared to the wildtype plants and the IF secondary cell wall thickness was increased in *bel1* relative to wildtype plants [21]. In our study, *PtrBEL1* was overexpressed in *PdOLP1*-OE poplar plants, which may suggest that the thinner xylem fibers and vessels of the cell walls of *PdOLP1*-OE plants may be caused by *PdOLP1* through upregulation of *PtrBEL1*. This result supports our hypothesis that overexpression of *PdOLP1* influences the expression of genes involved in secondary wall biosynthesis, which are coexpressed with *PdOLP1*.

Because secondary wall biosynthetic genes were repressed by *PdOLP1* in transgenic poplar and also regulated by transcription factors such as bHLH, BLH, and WRKY [21,57], we sought to determine whether secondary wall biosynthetic genes are regulated in through the transcription factor-PdOLP1 interaction. Transient expression assays in tobacco leaves were used, and we found that the *PdOLP1* promoter could be activated by PtobHLH7 and PtobZIP5 and repressed by PtoWRKY40 and PtoBLH8 (Figure 9b). Yan et al. [57] (2013) found that *S*. *bicolor SbbHLH1* overexpression in *Arabidopsis* caused downregulation of the lignin biosynthesis genes, which suggests that the bHLH transcription factor is a negative regulator of lignin biosynthesis in plants. In our research, PtobHLH7 was able to activate the promoter of *PdOLP1* by using the transcriptional activity assays. Therefore, we deduce that bHLH may regulate expression of *OLP* to influence the lignin biosynthetic genes in poplars.

Furthermore, direct evidence for the binding of these transcription factors to the promoter of the *PdOLP1* gene was obtained using a Y1H assay, which showed that PtoBLH8, PtoMYB3, and PtoWRKY40 could bind directly to the *PdOLP1* promoter (Figure 9c). Accordingly, there is reason to believe that secondary wall biosynthetic genes are regulated through the transcription factor-PdOLP1 interaction. Important transcription factors in wood formation such as PtoBLH8, PtoMYB3, and PtoWRKY40 can interact with *PdOLP1* and this interaction can further influence the expression of secondary wall biosynthetic genes in *PdOLP1*-overexpression or -knockdown lines, affecting secondary cell wall biosynthesis and wood development in poplar.

Taken together, this study provides new data to help understand the function of *PdOLP1* as a negative regulator of the lignin biosynthesis pathway and secondary wall formation in poplar. *PdOLP1* is an OLP that displays cytoplasmic localization and preferential expression in immature xylem and phloem tissues. Functional identification revealed that decreased expression of *PdOLP1* increases the number of cell layers and radial width in the xylem and phloem regions, increases the expression of genes involved in secondary cell wall biosynthesis, and thickens the fibers and vessels of the xylem cell walls in downregulated lines. Moreover, overexpression of *PdOLP1* resulted in thinner fibers and vessels of the cell walls in xylem cells. The coexpression network showed that *PdOLP1* cooperates with secondary wall biosynthesis genes, providing a molecular basis to reveal the role of *PdOLP1* participation in wood development. Furthermore, the *PdOLP1* promoter is activated or inhibited by transcription factors related to wood formation, such as PtobHLH7, PtoBLH8 and PtoWRKY40; PtoBLH8, PtoMYB3 and PtoWRKY40 can directly regulate transcription of the *PdOLP1* gene. This research may be valuable for cultivating new clones of tree species with an elevated wood yield and strength and improved bioenergy traits.

## 4. Materials and Methods

### 4.1. Plant Materials and Growth Conditions

Fifteen-year-old *Populus deltoides* plants were used to isolate the *PdOLP1* gene and analyze the gene expression pattern. Leaves, immature phloem, mature phloem, immature xylem, mature xylem, male flower buds and leaf buds were harvested three times from different regions of three *P. deltoides* plants for determination (Table S1). Hybrid poplar trees (*Populus alba* × *Populus glandulosa*) of clone 84K were used for genetic transformation experiments to characterize *PdOLP1* function. Branches from the current year of a 15-year-old *P. deltoides* tree were chosen for in situ hybridization.

### 4.2. In Situ Hybridization

The 30-bp *PdOLP1* nucleotide sequence was separately isolated and used for the synthesis of sense and antisense probes labeled with digoxigenin (DIG) following the DIG RNA Labeling mix instructions (Roche Diagnostics, Indianapolis, IN, USA). The tenth internodes of newly emerged branches of *P. deltoides* trees were sampled and fixed in FAA (formalin-acetic acid-alcohol) solution overnight. Stem specimens were embedded in paraffin wax and cut into 10-μm sections using a rotary Leica RM 6025 microtome (Leica Microsystems, Wetzlar, Germany) and hybridized with digoxigenin-labeled *PdOLP1* sense or antisense probes (Table S2). Hybridization signals were immunohistochemically detected using alkaline phosphatase-conjugated antibodies against digoxigenin. Images were obtained under an inverted fluorescence microscope (Carl Zeiss, Oberkochen, Germany).

### 4.3. Cloning of PdOLP1, Vector Construction and Genetic Transformation

Total RNA was extracted from the immature xylem (IX) of *P. deltoides* using RNeasy Plant Mini Kit (Qiagen, Valencia, CA, USA) as described in the manufacturer’s instructions. The full-length cDNA fragment encoding *PdOLP1* was amplified by PCR with gene-specific primers (Table S3) based on *PtOLP* (XM_024608084.1) from *P*. *trichocarpa* by PCR. The PCR products were subcloned into the pGEM-T Easy vector (Promega, Madison, WI, USA) and sequenced. The coding sequence (CDS) of *PdOLP1* with the stop codon was inserted into the expression vector pBI121-*ProNAC068-GUS* (GUS expression driven by the *ProNAC068* promoter was mainly detected in wood-forming tissues of poplar trees) in forward and reverse directions using the NEBuilder HiFi DNA Assembly master mix (New England BioLabs, E2621L) (the primers are listed in Table S3, Figure S1). These plant binary vectors (Figure S1) were transformed into *Agrobacterium tumefaciens* (strain GV3101) and subsequently transformed into hybrid poplar (*P*. *alba* × *P*. *glandulosa*) of clone 84K using the method described by Zhang et al. (2008) [79]. Rooted plantlets were acclimatized in a greenhouse at 25 °C under a 16-/8- h light/dark cycle.

### 4.4. Detection of Transgenic Plants

Transformed poplar plants were detected by PCR with gene-specific primers for the *NptII* gene (Table S3). Positive plants were further confirmed by Southern hybridization. Twenty-five micrograms of purified genomic DNA from transgenic and nontransformed poplar plants was digested with *Xba*I (New England Biolabs), followed by agarose gel electrophoresis and transfer to a nylon-based membrane (Hybond-N +, Amersham) using Vacuum Blotting Model 785 (BioRad, Hercules, CA, USA). The DNA fragment amplified from the *NptII* gene was labeled with biotin (BIO)-dUTP and used as a probe for hybridization [52]. Specific experimental methods followed the instructions of North2South Biotin Random Prime Kit and the chemiluminescent nucleic acid hybridization and detection kit (Pierce, Rockford, IL, USA).

### 4.5. Gene Expression Analyzed by Quantitative Real-Time PCR (qRT-PCR)

The level of *PdOLP1* expression in different 15-year-old *P. deltoides* tissues and in the stems of 1.5-year-old *PdOLP1* transgenic lines was detected by qRT-PCR using the primers listed in Table S3. qRT-PCR was performed using an ABI Prism 7500 sequence detector (Applied Biosystems, Foster City, CA, USA) with SYBR ^®^ Premix Ex TaqTM Kit (TaKaRa, Dalian, China); α-tubulin (*TUA1*) and ubiquitin (*UBQ1*) were used as control genes [80] for internal standardization of qRT-PCR data. Each PCR (final volume 20 μL) contained 1× SYBR Green PCR Master Mix, 200 nM of primers, and 1 μL of first-strand cDNA. Three replicates were conducted in parallel, and data analysis was performed following ABI Prism 7500 Sequence Detection System Users Guide. PCR primers for wood formation genes and genes coexpressed with *PdOLP1* were designed using Primer Premier 5.0 software (Premier Biosoft Int., Palo Alto, CA, USA), as listed in Table S4 and Table S5. Each gene was measured with three biological and three technical replicates, and error bars represent the standard error (SE) of the three replicates. The relative expression levels of target genes were analyzed using the 2−ΔΔCT method (the comparative Ct method) [81]. Standard curves were analyzed to compare the gene-specific PCR efficiency from 10-fold series dilutions of the mixed cDNA templates for each primer pair. Slope values and correlation coefficients (R^2^) were calculated from the standard curve, and the PCR amplification efficiencies (E) were analyzed by the following formula: E = (10^−1/slope^-1) × 100 [82].

### 4.6. Subcellular Localization

The full-length coding sequence (CDS) of *PdOLP1* without the termination codon was amplified and ligated to the N-terminus of GFP driven by the cauliflower mosaic virus (*CaMV*) 35S promoter. The vector 35S-GFP was transformed simultaneously in parallel as a control. All of these constructs were transformed into onion epidermal cells via particle bombardment [83]. The onion epidermis was mounted onto glass slides. For the DNA construct, 2 µg of DNA was mixed with 50 µL of 2.5 M CaCl_2_, 25 µL of 0.1 M spermidine, and 50 µL of gold microcarrier, vortexed vigorously for 2.5 min, and centrifuged at 10,000 rpm (5810R, Eppendorf, Germany) in a microcentrifuge for 15 s. The pellet was washed with 180 µL of ethanol and then resuspended in 30 µL of ethanol. The DNA-gold particles were bombarded into cells at a pressure of 1,100 lb/in2 using the Biolistic Particle Delivery System 1000/He (Bio-Rad). The nuclei of of the transfected onion cells were stained with 4′,6-diamidino-2-phenylindole (DAPI), and GFP fluorescent signals were detected using an inverted fluorescence microscope (Carl Zeiss, Oberkochen, Germany).

### 4.7. Histological Analysis and Microscopy

The 15th internodes on the stems of 1.5-year-old poplar plants were fixed in FAA buffer and then embedded in paraffin. The stem segments were cut into 8-μm-thick sections with a microtome (Leica Microsystems, Wetzlar, Germany) and stained with 0.05% (*w*/*v*) toluidine blue O or stained for lignin with phloroglucinol-HCl, followed cby observation under an inverted fluorescence microscope (Carl Zeiss, Oberkochen, Germany). Lignin autofluorescence was also assessed under a confocal laser microscope (Zeiss, Jena, Germany). We used 0.2-cm-thick stem sections of *PdOLP1* transgenic and nontransgenic lines for scanning electron microscopy (S-4800, HITACHI, Tokyo, Japan). Secondary wall thickness of vessels and fibers was measured, and at least three transformed lines were measured.

### 4.8. Microfibril Angle Measurement

We measured the angles of stem blocks of 1.5-year-old transformed and non-transformed poplar lines at 5 cm above the pot. The microfibril angle (MFA) was measured as described previously [84]. Glacial acetic acid/hydrogen peroxide solution (1:1, *v*/*v*) was used to dissociate the fibers of different samples at 60 °C overnight, and polarized microscopy was used to measure individual fibers of each sample (Olympus BX51; Olympus, Melville, NY, USA).

### 4.9. Determination of Lignin Content, Wood Density, Total Biomass, and Carbon Storage

Lignin content of 1.5-year-old transformed and non-transformed poplar plants was evaluated using dry extract-free cell wall residue (CWR), which was ground to powder and passed through a 0.5-mm sieve before solvent extraction (2:1 [*v*/*v*] toluene:ethanol, ethanol and then water). The lignin contents of basal stem segments from transgenic and nontransgenic plants were measured according to the Klason procedure [85]. Three biological replicates were performed. Drainage method is used to measure volume, drying method is used to measure wood quality and total biomass, and basic density is determined by wood quality/wood volume. The specific steps refer to the methods of national standards of *GB/T 1933–2009 Determination of wood density*. We crushed, mixed, and dried the sample, and 2mg was used for testing of carbon storage by using Vario EL cube element analyzer. For detailed methods as described previously [86].

### 4.10. Phylogenetic Analysis and Statistical Analyses

The CDSs of OLPs were identified from the website (available online: http://www.phytozome.com, accessed on: 6 June 2019) and the Swedish Center for Tree Functional Genomics project database (Populus DB). Multiple sequence alignments were generated with the Clustal W program (available online: www.ebi.ac.uk/clustalw/, accessed on: 10 June 2019). The phylogenetic relationships of OLPs were performed using MEGA version 7.0 [87] by the neighbor-joining method. Measurement data for plant height, cell wall thickness, and lignin content, etc, were subjected to statistical analysis using one-way analysis of variance. Quantitative differences between two groups of data in all comparisons were statistically significant (*p* < 0.05; ANOVA, Fisher test).

### 4.11. Transient Expression Assay

Promoter fragments of the *PdOLP1* gene were amplified by PCR using the gene-specific primers listed in Table S3. The amplified fragments were fused to the GUS reporter gene in the modified pCAMBIA 1305 vector to generate reporter constructs. To create the effector constructs, the PtoWRKY40, PtoARF1, PtobZIP5, PtoBLH8, and PtobHLH7 coding regions were amplified from poplar (*Populus tomentosa*) xylem cDNA (primers in Table S3) and ligated between the 35S promoter and the NOS terminator after removing GUS from the pBI121 vector. Tobacco (*Nicotiana benthamiana*) leaves were injected with *Argrobacterium* cells containing the reporter and effector plasmids [88]. Briefly, *A. tumefaciens* GV3101 carrying the constructed plasmids was cultured to OD600 = 0.5; the samples were combined at equal volumes, incubated at room temperature without shaking for 3 h and infiltrated into *N*. *benthamiana* leaves. The GUS expression level was measured by spectrophotometry [89]. In each experiment, the expression level of the GUS reporter gene in *N*. *benthamiana* leaves transfected with the reporter construct alone was used as the control. Data are presented as the mean ± SE of three independent experiments.

### 4.12. Yeast One-Hybrid Assay

The yeast one-hybrid assay was performed by using Matchmaker Gold Yeast One-Hybrid System (Clontech, http://www.clontech.com/, accessed on: 3 January 2020) following the manufacturer’s instructions. The promoter sequence of the *PdOLP1* gene was ligated to the vector pAbAi (Y1H Gold), and the plasmid was linearized and integrated into the yeast genome. Yeast cells were then transformed with the pGADT7-AD vector (Y1H Gold) carrying the PtoWRKY40, PtoARF1, PtobZIP5, PtoMYB3, PtoBLH8, or PtobHLH7 coding sequence. The protein–DNA interaction was determined based on the ability of the transformed yeast to grow on SD/-Leu/-Ura medium with Aureobasidin A (AbA) according to the manufacturer’s protocol.

### 4.13. Coexpression Network Analysis

The coexpression data for *PdOLP1* were obtained from Phytozome (available online: https://phytozome.jgi.doe.gov/pz/portal.html, accessed on: 15 July 2019). For *Populus* genome-wide coexpression network construction, transcriptome data from 24 various *P*. *trichocarpa* tissues [90] were used. Genes with Pearson correlation coefficients >0.85 were selected to build the coexpression network of *PdOLP1*. The network diagram was generated according to Cytoscape software [91] (The Cytoscape v1.1 Core was downloaded from http://www.cytoscape.org/, accessed on: 27 July 2019).

## Figures and Tables

**Table 1:** Genes related to wood formation in the *PdOLP1* coexpression network.

Gene Name	Gene ID	Arabidopsis Orthologs	Description	Reference
PtrXYL5	Potri.018G009300	AT4G31590	Poplar glycosyltransferase is involved in the process of wood formation.	[57]
PtrbHLH110	Potri.005G230800	AT1G27660	The bHLH transcription factor regulates early xylem development.	[59]
PtrbHLH71	Potri.005G071100	AT3G24140		
PtrSER6	Potri.014G026100	AT4G02630	The protein kinase family protein is associated with cell wall formation.	[60,61]
PtrZF4	Potri.008G055100	AT5G02460	Zinc finger protein inducing secondary cell wall thickening in stem.	[62]
PtrGLY48	Potri.005G058300	AT2G13680	Glycosyl transferases participate in cell wall biosynthesis.	[63]
PtrPK9	Potri.001G117800	AT4G22730	Leucine-rich-repeat receptor-like kinase associated with fiber development and secondary cell wall formation.	[64]
PtrLRR5	Potri.003G179100	AT1G80630	A calmodulin-binding proteins involved in the differentiation of tracheary elements.	[65]
PtrPOD3	Potri.003G214700	AT5G06720	Peroxidase is responsible for cell wall lignification.	[58,66,67]
PtrPCD2	Potri.011G001800	AT4G21890	Differentiation of procambial or cambial cells to tracheary elements is a typical example of programmed cell death in higher plants.	[68,69]
PtrGRAS5	Potri.003G202100	AT1G50420	GRAS proteins are required for maintenance of shoots and root indeterminacy.	[70,71]
PtrGLU2	Potri.006G116900	AT5G03760	Glucomannan-synthases are believed to have a role in hemicellulose biosynthesis.	[72]
PtrPP2	Potri.012G120400	AT4G19840	Several phloem proteins are cell-wall proteins.	[32]
PtrNAC75	Potri.018G068700	AT4G29230	NACs are involved in the regulation of secondary cell wall biosynthesis.	[18,73]
PtrBEL1	Potri.010G197300	AT5G02030	BEL1 interacts with proteins related to wood formation to regulate secondary cell wall formation.	[21]
PtrbZIP42	Potri.017G106700	AT3G30530	BZIPs show vascular cell expression patterns and can bind to a regulatory element that controls xylem-specific gene expression.	[74]
PtrMYB12	Potri.002G168900	AT5G53200	MYB is involved in the regulation of secondary cell wall biosynthesis.	[75,76]
PtrMYB308	Potri.018G005300	AT5G35550		
PtrDyn8	Potri.003G024900	AT1G60500	Dynamin is essential for proper secondary cell wall synthesis.	[77,78]

## Supplementary Materials

Supplementary materials can be found at https://www.mdpi.com/1422-0067/21/11/3993/s1.
