# Supplementary material for: The Osmotin-Like Protein Gene PdOLP1 Is Involved in Secondary Cell Wall Biosynthesis during Wood Formation in Poplar
Source: Int J Mol Sci. 2020 Jun 2;21(11):3993. doi: 10.3390/ijms21113993 (PMC7312728; doi:10.3390/ijms21113993)
Supplement: Supplementary file 1 [file ijms-21-03993-s001.pdf]

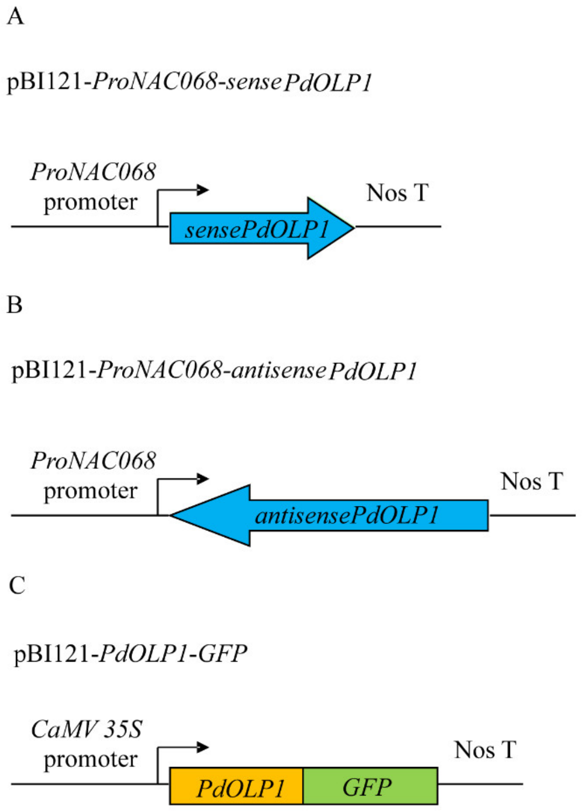

Figure S1. Recombinant vector map of various constructs used for genetic transformation. (A) Construction of the pBI121-*ProNAC068*-sense *PdOLP1* vector overexpressing the poplar *PdOLP1* gene; (B) Construction of the pBI121-*ProNAC068*-antisense *PdOLP1* vector expressing the antisense poplar *PdOLP* gene; (C) Construction of the pBI121-*PdOLP1-GFP* vector expressing the PdOLP1-GFP fusion protein.

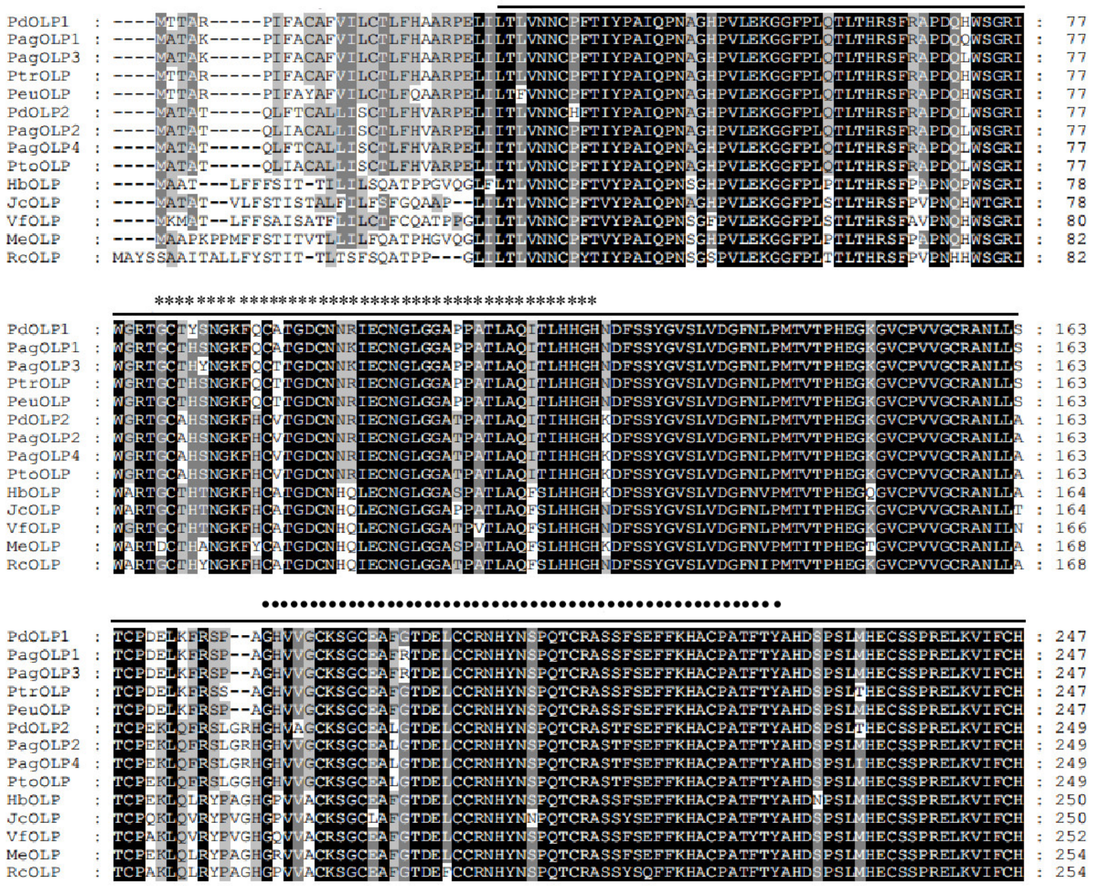

Figure S2. ClustalW alignment of the amino acid sequences of PdOLP1, and its homologous proteins.

The GenBank accession number for PdOLP1: MK052942; PdOLP2: Podel.01G248000.1; PagOLP1: Pop_G09G014129.T1; PagOLP2: Pop_A01G056866.T1; PagOLP3: Pop_A09G059600.T1; PagOLP4: Pop_G01G020795.T1; PtrOLP: XP_024463852.1; PeuOLP: XP_011042680.1; PtoOLP: APA20308.1; MeOLP: XP_021626825.1; HbOLP:XP_021636655.1; RcOLP: XP_002531364.1; VfOLP: ARV78462.1; JcOLP: XP_012065012.1. “*” is used to indicate the AWS domain, “-” indicates the THN domain, and the “.” means the FU domain, which involved in the mechanism of signal transduction by receptor tyrosine kinases.

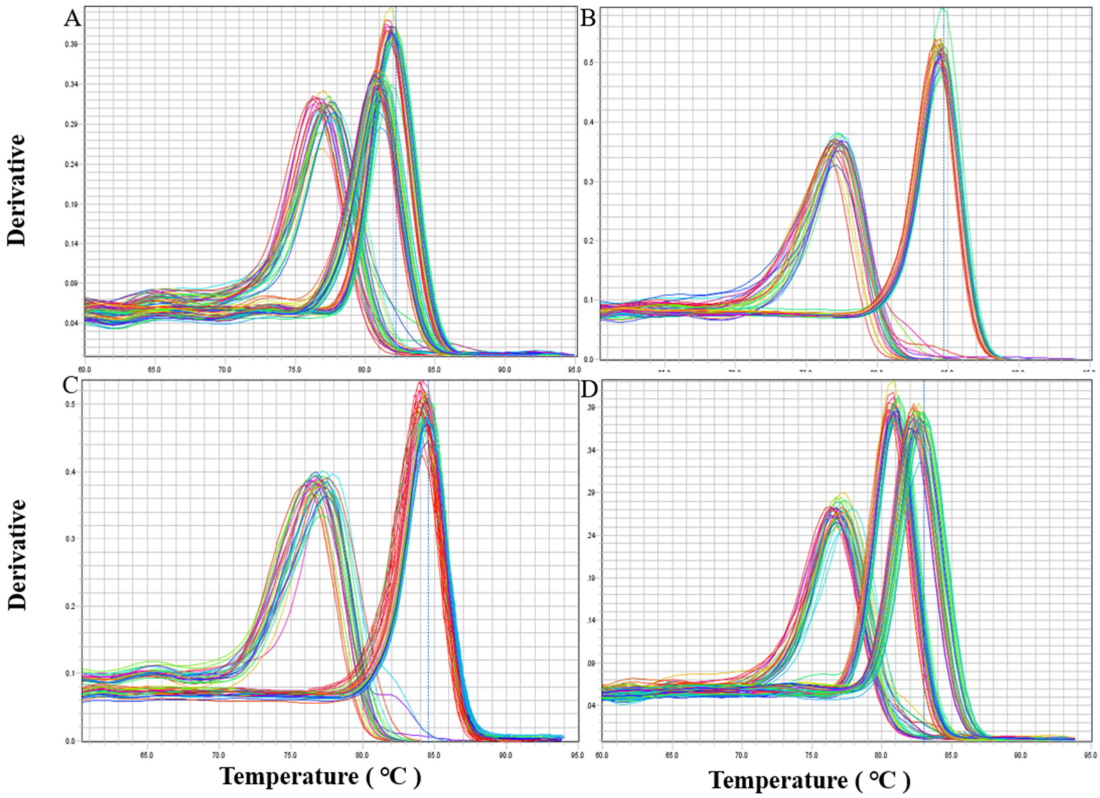

Figure S3. Melting curves of eight secondary cell wall biosynthesis genes. The left, middle and right melting curves of figure A represents the tested genes *PtrFRA1*, *PtrC4H1* and *PtrCCoAOMT1*, respectively.
The left and right melting curves of figure B represents the tested genes *TUA1* and *PtrCAD10*, respectively.
The left and right melting curves of figure C represents the tested genes *PtrTUB7* and *PtrMYB90*, respectively.
The left, middle and right melting curves of figure D represents the tested genes *UBQ1*, *PtrSuS1* and *Ptr4CL3*, respectively.

Figure S4. Standard curves of eight secondary cell wall biosynthesis genes.

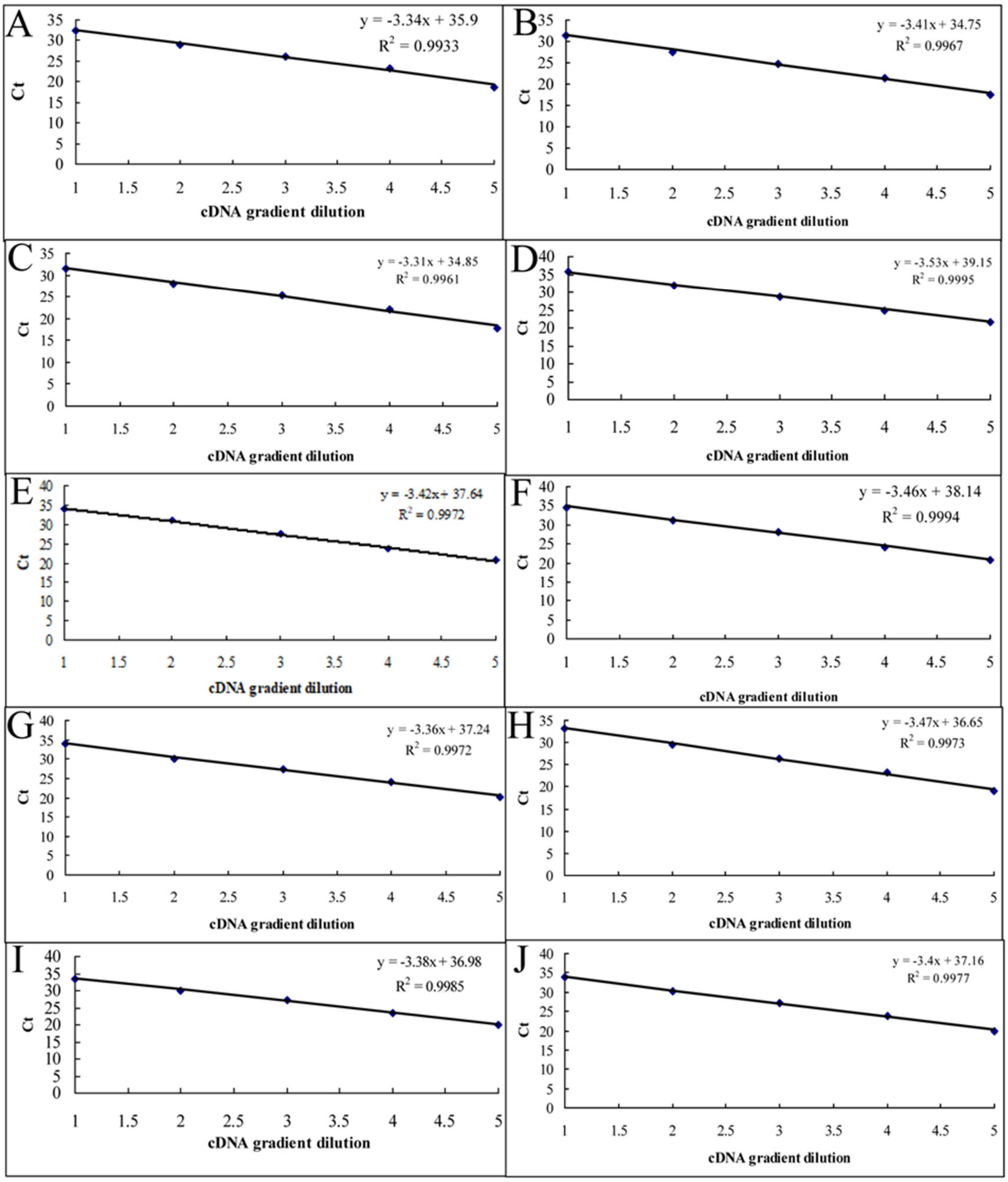

Figure A shows the standard curve of *PtrC4H1*.
Figure B shows the standard curve of *PtrCAD10*.
Figure C shows the standard curve of *PtrCCoAOMT1*.
Figure D shows the standard curve of *Ptr4CL3*.
Figure E shows the standard curve of *PtrMYB90*.
Figure F shows the standard curve of *PtrSuS1*.
Figure G shows the standard curve of *PtrTUB7*.
Figure H shows the standard curve of *PtrFRA1*.
Figure I shows the standard curve of *TUA1*.
Figure J shows the standard curve of *UBQ1*.

Table S1. Details of the samples isolated from *Populus deltoides*.

Name	Description	Collection Date
**Immature xylem**	Collected from a vigorously developing *Populus deltoides* Marsh tree in a field near the Chinese Academy of Forestry. Bark was peeled and four to eight partially lignified cell layers were shaved off from the wood side at chest height. Samples were scraped into liquid nitrogen.	2009/8/20
**Immature phloem**	The bark was peeled and four to eight cell layers from the bark side representing immature phloem were scraped into liquid nitrogen.	2009/8/20
**Mature phloem**	Isolated from the bark side, nearer to the pith than immature phloem. Exists inside of and is stiffer than immature phloem. Scraped into liquid nitrogen.	2009/8/20
**Mature xylem**	Isolated from the exposed wood core, closer to the pith. Most rigid vascular tissue. Scraped into liquid nitrogen.	2009/8/20
**Leaf bud**	Very short internodes of the central branches (not apex), not the developmental branches. Initiate from leaf primordia and form branches and mature leaves after blooming.	2009/8/20
**Male flower bud**	Terminal buds of shoot apex/tips of a 15-year-old adult male tree. Prerequisite for floral/flower initiation or differentiation during the growth stage.	2009/8/20

Table S2. Sequences of the sense and antisense probes used for in situ hybridization.

**Antisense RNA probe (5′-3′)**	**Sense RNA probe (5′-3′)**
CGTGGTGGAGGGTAATCTGTGCCAAAGCGG	CCGCTTTGGCACAGATTACCCTCCACCACG

Table S3. Primers used for vector construct and expression analysis.

**Primer name**	**Forward primer** (5′-3′)	**Reverse primer** (5′-3′)
P1	ATGACTACTGCTAGGCCAAT	TTAGTGACAAAAGATAACTTTA
P2	CTAGAGTCGACCTGCAGACGGCCCGGGCTGGTATCC	CCTCAGATCTACCATGGTTTTCCTTTTGGTTCAAGA G

P3	CATTTGGAGAGAACACGGGGGACTCTAGAATGACTACT GCTAGGCCAATCT	TGTTTGAACGATCGGGGAAATTCGAGCTCTTAGTGA CAAAAGATAACCTTAAG
P4	CATTTGGAGAGAACACGGGGGACTCTAGATTAGTGACA AAAGATAACCTTAAG	TGTTTGAACGATCGGGGAAATTCGAGCTCATGACTA CTGCTAGGCCAATCT
P5	TCCGGCCGCTTGGGTGGAGAG	CTGGCGCGAGCCCCTGATGCT
P6	GCCCTGTGGTAGGTTGC	TGCGGACTGTTGTAATGGTT
P7	AGGTTCTGGTTTGGGGTCTT	TTGTCCAAAAGCACAGCAAC
P8	GTTGATTTTTGCTGGGAAGC	GATCTTGGCCTTCACGTTGT
P9	ATGGATAACTCATCCTGGGTT	TCACCACTTTGTGTGATTTTGC
P10	ATGGCATTTGCGGCTATGAATC	TCAGCATCCAGGTCCAATAGT
P11	ATGCACGAGGATTACAAGTG	TCATGTTCTAAGTAGCTCTGC
P12	ATGCATATGGCTACCTATTATAC	TCAAGCTACGAAATCATGAAAC
P13	ATGTCTCTCGACTGTCTACGCT	TTAGGTCATGCATTAAGTAGGAC
P14	GGCCTCTAGAATGACTACTGCTAGGCCAAT	GGCGGTACCTTAGTGACAAAAGATAACTTTA

Note:

(1) Primer pair of P1 for amplifing the coding region of *PdOLP1* from cDNA of *P. deltoides,* and Primer of P2 for amplifing the promoter fragments of *PdOLP1* from *P. deltoides*;
(2) Primer pair of P3 and P4 for amplifing the sense and antisense *PdOLP1* from cDNA of *P. deltoides*;
(3) qRT-PCR primers P5 for *NptII* gene, P fo6r *PdOLP1*, P7 and P8 for *TUA1* and *UBQ1*;
(4) Primer pair used for amplifing the coding region of PtoWRKY40 (P9), PtoARF1 (P10), PtobZIP5 (P11), PtoBEL8 (P12), and PtobHLH7 (P13) of *P. tomentosa*;
(5) Primer of P14 for amplifing the *PdOLP1* and inserted into pGreenII 62-SK vector.

Table S4. Primers used for analysis of wood formation genes.

**Gene name**	Description	NCBI or JCI accession number	Primer sequence (forword/reverse)	Amplification efficiency (%)	R^2
PtrCAD10	Cinnamyl alcohol dehydrogenase	XM_002300175.1	CAGCACTTTGTACTCCGTATTCC/TGCTTCCCTGGTTCTGTCATT	95.89	0.9967
Ptr4CL3	4-coumaryl:CoA ligase	XM_002297663.2	ACTAGCCCATCCAGAGATATCCGA/TCATCTTCGGTGGCCTGAGACTTT	92.16	0.9995
PtrC4H1	4-coumarate:cinnamate-4-hydroxylase	AF302495.1	CCCTCTTGGGTTCTTTCGTT/CAAACACGGGGACAGGTATA	99.07	0.9933
PtrMYB90	Myeloblastoma 90	XM_002329715	CCGAAGAGGAAGAAGAAAGACT/TCAGTGCGACCTGGGAAAA	95.85	0.9972
PtrCCoAOMT1	Caffeoyl CoA 3-O-methyltransferase	XM_002313089	TTGGTGGGCTGATTGGGTA/GCTCCAAAACAAAGTCCCTGT	100.005	0.9961
PtrTUB7	beta-tubulin 7	XM_002299006.1	TTGAGCCATACAACGCCAC / CGGAAGCAGATGTCATACAAA	98.61	0.9972
PtrFRA1	Fragile fiber 1	XM_002302396.1	CAGCAGAACTGTTATGATAGC / TGTTGCGGGCACGATTTG	94.13	0.9973
PtrSuS1	Sucrose synthase 1	GU559729.1	TTTCCCTCGCCCAACTCTT/GATGCAGGCTTTCCTTGTCA	94.55	0.9994
TUA1	α-tubulin 1	CA822230;CA825391	AGGTTCTGGTTTGGGGTCTT/TTGTCCAAAAGCACAGCAAC	97.7	0.9985
UBQ1	Ubiquitin 1	BU879229	GTTGATTTTTGCTGGGAAGC/GATCTTGGCCTTCACGTTGT	95.92	0.9977

Table S5 Primers used in qRT-PCR analysis of *PdOLP1* coexpressed genes

**Gene name**	**Gene ID**	**Forward primers (5'→3')**	**Reverse primers (5'→3')**
PtrXYL5	Potri.018G009300	CCGAGAAGTTAGGCAAAG	AACCCAAACAAAGCACC
PtrBHLH110	Potri.005G230800	AATAACAGAGGCTAAAAGG	AAAAGGAGGGCAGGGAGA
PtrBHLH71	Potri.005G071100	GCATCAATCATAGGTGGGGC	TCCGAGAAGAGAGAAGAGCA
PtrSER	Potri.014G026100	CAACTCGTGTAATGGGAAC	AGGGCGGCTGTAATCAAC
PtrZF	Potri.008G055100	GGTTCAACTACCTGAAACAGC	CTTCTACAGCCACCGCCAACA
PtrGLY48	Potri.005G058300	TGCTTTTCTCTTGGTTGGCC	GTGCCAAAAGGTTCGTGTCT
PtrPK	Potri.001G117800	GCCATTGTTGCAGGGGTTAT	TGCTTAGCCGACCATCAGAA
PtrLRR5	Potri.003G179100	AATGGAGTGGTGCAAAGCTG	GGGATGGCCTTGAAAACACC
PtrPOD	Potri.003G214700	TGTTCGTTTGGCAGGAGG	AAGGGCAACCAGGTCACTA
PtrPCD2	Potri.011G001800	ACCCTCGTGATTCCCCTTTT	GATAATGGCAGCGACCGAAG
PtrGRAS	Potri.003G202100	TTCACCGAAGATGAGCAGCT	AGCTTTTGTGACCCTCTCCA
PtrGLU	Potri.006G116900	GCGCACCATTCCCTTTCTAG	GCCCACTTCCTGTTCAACTG
PtrPP2	Potri.012G120400	TCACTGTGGATGTGGCAGA	TTTGGGAGAACCAGTGAG
PtrNAC75	Potri.018G068700	AGGTCTGCATCTGGTTTGGA	TCAACCACTCTGCCTCTTGT
PtrBEL1	Potri.010G197300	CTGCTGGTGTTAGTGGAAGC	TTGACGTGGTGAGGTATGCT
PtrBZIP42	Potri.017G106700	CACAGGTGGTTTGGTTCCG	CAGGTGATGTCTTCCAGGTC
PtrMYB	Potri.002G168900	TGATTTGGTAGAGGTAAGCAGC	CCTGTCTCCGACCAGCTTAT
PtrMYB308	Potri.018G005300	AGCGTGGACACCTCTTGAAG	ATTCATCCAACGAAGCCTGC
PtrDyn	Potri.003G024900	GCTCAACCAATACTCAGGCG	CGGGGAAGGAAATTTGGCAA

Table S6. OLP sequences of *P. deltoides* and the homologous genes from 51 other plants.

>PdOLP1 MK052942 Populus deltoides
MTTARPIFACAFVILCTLFHAARPELILTLVNNCPFTIYPAILPNAGHPVLEKGGFPL
QTLTHRSFRAPDQHWSGRIWGRTGCTYSNGKFQCATGDCNNRIECNGLGGAPPAA
LAQITLHHGHNDFSSYGVSLVDGFNLPMTVTPHEGKGVCPVVGCRANLLSTCPDE
LKFRSPAGHVVGCKSGCEAFGTDELCCRNHYNSPQTCRASSFSEFFKHACPATFTY
AHDSPSLMHECSSPRELKVIFCH

>PdOLP2 Podel.01G248000.1 Populus deltoides
MATATQLFTCALLISCTLFHVARPELIITLVNNCHFTIYPAIQPNAGHPVLEKGGFPL
QTLTHRSFRAPDQLWSGRIWGRTGCAHSNGKFHCVTGDCNNRIECNGLGGATPAT
LAQITIHHGHKDFSSYGVSLVDGFNLPMTVTPHEGKGVCPVVGCRANLLATCPEKL
QFRSLGRHGHVAGCKSGCEALGTDELCCRNHYNSPQTCRASTFSEFFKHACPATFT
YAHDSPSLTHECSSPRELKVIFCH

>PagOLP1 Pop_G09G014129.T1 Populus alba × Populus glandulosa
MATAKPIFACAFVILCTLFHAARPELILTLVNNCPFTIYPAIQPNAGHPVLEKGGFPL
QTLTHRSFRAPDQQWSGRIWGRTGCTHSNGKFQCATGDCNNKIECNGLGGAPPAT
LAQITLHHGHNDFSSYGVSLVDGFNLPMTVTPHEGKGVCPVVGCRANLLSTCPDE
LKFRSPAGHVVGCKSGCEAFRTDELCCRNHYNSPQTCRASSFSEFFKHACPATFTY
AHDSPSLMHECSSPRELKVIFCH

>PagOLP2 Pop_A01G056866.T1 Populus alba × Populus glandulosa
MATATQLIACALLISCTLFHVARPELIITLVNNCPFTIYPAIQPNAGHPVLEKGGFPL
QTLTHRSFRAPDQLWSGRIWGRTGCAHSNGKFHCVTGDCNNRIECNGLGGATPAT
LAQITIHHGHKDFSSYGVSLVDGFNLPMTVTPHEGKGVCPVVGCRANLLATCPEKL
QFRSLGRHGHVVGCKSGCEALGTDELCCRNHYNSPQTCRASTFSEFFKHACPATFT
YAHDSPSLMHECSSPRELKVIFCH

>PagOLP3 Pop_A09G059600.T1 Populus alba × Populus glandulosa
MATAKPIFACAFVILCTLFHAARPELILTLVNNCPFTIYPAIQPNAGHPVLEKGGFPL
QTLTHRSFRAPDQLWSGRIWGRTGCTHYNGKFQCTTGDCNNKIECNGLGGAPPAT
LAQITLHHGHNDFSSYGVSLVDGFNLPMTVTPHEGKGVCPVVGCRANLLSTCPDE
LKFRSPAGHVVGCKSGCEAFRTDELCCRNHYNSPQTCRASSFSEFFKHACPATFTY
AHDSPSLMHECSSPRELKVIFCH

>PagOLP4 Pop_G01G020795.T1 Populus alba × Populus glandulosa
MATATQLFTCALLISCTLFHVARPELIITLVNNCPFTIYPAIQPNAGHPVLEKGGFPL
QTLTHRSFRAPDQLWSGRIWGRTGCAHSNGKFHCVTGDCNNRIECNGLGGATPAT
LAQITIHHGHKDFSSYGVSLVDGFNLPMTVTPHEGKGVCPVVGCRANLLATCPEKL
QFRSLGRHGHVVGCKSGCEALGTDELCCRNHYNSPQTCRASTFSEFFKHACPATFT
YAHDSPSLIHECSSPRELKVIFCH

>PtrOLP XP_024463852.1 Populus trichocarpa
MTTARPIFACAFVILCTLFHAARPELILTLVNNCPFTIYPAIQPNAGHPVLEKGGFPL
QTLTHRSFRAPDQHWSGRIWGRTGCTHSNGKFQCTTGDCNNRIECNGLGGAPPAT
LAQITLHHGHNDFSSYGVSLVDGFNLPMTVTPHEGKGVCPVVGCRANLLSTCPDE
LKFRSSAGHVVGCKSGCEAFGTDELCCRNHYNSPQTCRASSFSEFFKHACPATFTY
AHDSPSLTHECSSPRELKVIFCH

>PeuOLP XP_011042680.1 Populus euphratica
MTTARPIFAYAFVILCTLFQAARPELILTFVNNCPFTIYPAIQPNAGHPVLEKGGFPL
QTLTHRSFRAPDQHWSGRIWGRTGCTHSNGKFQCTTGDCNNRIECNGLGGAPPAT
LAQITLHHGHNDFSSYGVSLVDGFNLPMTVTPHEGKGVCPVVGCRANLLSTCPDE
LKFRSPAGHVVGCKSGCEAFGTDELCCRNHYNSPQTCRASSFSEFFKHACPATFTY
AHDSPSLMHECSSPRELKVIFCH

>PtoOLP APA20308.1 Populus tomentosa
MATATQLIACALLISCTLFHVARPELIITLVNNCPFTIYPAIQPNAGHPVLEKGGFPL
QTLTHRSFRAPDQLWSGRIWGRTGCAHSNGKFHCVTGDCNNRIECNGLGGATPAT
LAQITIHHGHKDFSSYGVSLVDGFNLPMTVTPHEGKGVCPVVGCRANLLATCPEKL
QFRSLGGHGHVVGCKSGCEALGTDELCCRNHYNSPQTCRASTFSEFFKHACPATFT
YAHDSPSLMHECSSPRELKVIFCH

>MeOLP XP_021626825.1 Manihot esculenta
MAAPKPPMFFSTITVTLLILFQATPHGVQGLILTLVNNCPFTVYPAIQPNSGHPVLEK
GGFPLPTLTHRSFPAPNQHWSGRIWARTDCTHANGKFYCATGDCNHQLECNGLGG
ASPATLAQFSLHHGHKDFSSYGVSLVDGFNVPMTITPHEGTGVCPVVGCRANLLAT
CPEKLQLRYPAGHGRVVACKSGCEAFGTDELCCRNHYNSPQTCRASSFSEFFKHAC
PATFTYAHDSPSLMHECSSPRELKVIFCH

>HbOLP XP_021636655.1 Hevea brasiliensis
MAATLFFFSITTILILSQATPPGVQGLFLTLVNNCPFTVYPAIQPNSGHPVLEKGGFPL
PTLTHRSFPAPNQPWSGRIWARTGCTHTNGKFHCATGDCNHQLECNGLGGASPAT
LAQFSLHHGHKDFSSYGVSLVDGFNVPMTVTPHEGQGVCPVVGCRANLLATCPEK
LQLRYPAGHGPVVACKSGCEAFGTDELCCRNHYNSPQTCRASSFSEFFKHACPATF
TYAHDNPSLMHECSSPRELKVIFCH

>RcOLP XP_002531364.1 Ricinus communis

MAYSSAAITALLFYSTITTLTSFSQATPPGLILTLVNNCPYTIYPAIQPNSGSPVLEKG
GFPLTTLTHRSFPVPNHHWSGRIWARTGCTHYNGKFHCATGDCNHQIECNGLGGA
TPATLAQFSLHHGHNDFSSYGVSLVDGFNIPMTVTPHEGKGVCPVVGCRANLLAT
CPAKLQLRYPAGHGPVVACKSGCEAFGTDEFCCRNHYNSPQTCRASSYSQFFKHA
CPATFTYAHDSPSLMHECSSPRELKVIFCH

>DzOLP XP_022756598.1 Durio zibethinus
MASPLYFSLFFLATLFLLCSFAKASEPDLILTLVNNCPFTIYPAIQPNAGHPVLQRGG
FALQTLTHRSFPAPTVHWSGRIWARTGCTHSNGQFYCATGDCGHRIECNGLGGAT
PVTLAQFSLHHRGHKDLSSYEVSLVDGFNIPMTVTPHDGKGLCPVVGCRANLLAT
CPNQLQLRSPPGHGPVVGCKSGCQAFGTDELCCRNHYNSPQTCRASSYSEFFKHAC
PATFTYAHDSPSLMHDCASPRELKVIFCH

>CjOLP BAI63297.1 Citrus jambhiri
MASTSPIFISATILLLLLSSYAKASFILTLVNNCPFPVWPAIQPNAGHPVLERGGFYLHS
LTHRSFPAPAQHWSGRIWARTGCTYAHGKFSCVTGDCGHRLESNGLGGATPATLA
QFSLHHGHNDLSSYGVSLVDGFNVPMTVTPHEGKGVCPVVGCRADLLATCPQNL
QVRSPPGHGRVVACKSGCEAFGTDELCCRNHYNSPQTCRASSFSEFFKHACPATMT
YAHDSPSLMHDCSSPRELKVIFCH

>VfOLP ARV78462.1 Vernicia fordii
MKMATLFFSAISATFLILCTFCQATPPGLILTLVNNCPFTVYPAIQPNSGFPVLEKGG
FPLSTLTHRSFAVPNQHWSGRIWGRTGCTHTNGKFHCATGDCNHQLECNGLGGAT
PVTLAQFSLHHGHNDFSSYGVSLVDGFNLPMTVTPHEGKGVCPVVGCRANILNTC
PAKLQVRYPVGHGQVVACRSGCEAFGTDELCCRNHYNSPQTCRASSFSEFFKHAC
PATYTYAHDSPSLMHECSSPRELKVIFCH

>TcOLP XP_007016468.1 Theobroma cacao
MASPLCASLFCFATLFLLCSFAAATQPGLILTLVNNCPFTVYPAIQPNAGHPVLERG
GFALQTLTHRSFPAPTAHWSGRIWARTGCTHSNGHFYCATGDCGHRIECNGLGGA
TPVTLAQFSLHHGGHKDLSSYAVSLVDGFNVPMTVTPHEGKGLCPVVGCRANLLA
TCPDKLQLRSPPGHGPVVGCKSGCEAFGTDELCCRNHYNSPQTCRASSFSEFFKHA
CPATFTYAHDSPSLTHECASPRELKIIFCH

>CsiOLP XP_006488293.1 Citrus sinensis

MASTSPIFISATILLLLLSSYAKASFILTLVNNCPFPVWPAIQPNAGHPVLERGGFYLHS
LTHRSFPAPAQHWSGRIWARTGCTYAHGKFSCVTGDCGHRLECNGLGGATPATLA
QFSLHHGHNDLSSYGVSLVDGFNVPMTVTPHEGKGVCPVVGCRADLLATCPQNL
QVRSPPGHGRVVACKSGCEAFGTDELCCRNHYNSPQTCRASSFSEFFKHACPATMT
YAHDSPSLMHDCSSPRELKVIFCH

>CcOLP XP_006424795.1 Citrus clementina
MASTSPIFISATILLLLLSSYAKASFILTLVNNCPFPVWPAIQPNAGHPVLERGGFYLHS
LTHRSFPAPAQHWSGRIWARTGCTYAHGKFSCVTGDCGHRLECNGLGGATPATLA
QFSLHHGHNDLSSYGVSLVDGFNVPMTVTPHEGKGVCPVVGCRADLLATCPQNL
QVRSPPGHGRVVACKSGCEAFGTDELCCRNHYNSPQTCRASSFSEFFKHACPATMT
YAHDSPSLMHDCSSPRELKVIFCH

>VvOLP XP_002281193.1 Vitis vinifera
MASSSTISSSFVFFAVFLFLCTLSHATNPGLILTVVNNCPFTIWPAIQPNAGHDVLER
GGFALHTLTHRSFAAPSHHWSGRIWARTGCTYINGKFSCVTGDCGGRLECSGSGG
AAPATLAQFTLHHGQNDFSSYAVSLVDGFNLPMTVTPHEGKGLCPVVGCRANLLE
TCPGKLQMWSPPEHGKVVACKSGCEAFGTDELCCRNHYNSPQTCRASSFSEFFKH
ACPATFTYAHDSPSLMHECSSPRELKVIFCH

>HuOLP XP_021278215.1 Herrania umbratica
MASTLCASLFCFVTLFLLCSFAGATQPGLILTLVNNCPFTVYPAIQPNAGHPVLERG
GFALQTLTHRSFPAPTAHWSGRIWARTGCTHSNGHFYCATGDCGHRIECSGLGGA
PPVTLAQFSLHHGGHKDLSSYAVSLVDGFNVPMTVTPHEGKGLCPVVGCKANLLA
TCPDKLQLRSPPGHGPVVGCKSGCQAFGTDELCCRNHYNSPQTCRASSFSEFFKHA
CPATFTYAHDSPSLTHECASPRELKIIFCH

>JrOLP XP_018823302.1 Juglans regia
MLFSRVKKMASLSLRFTLFSTAAFLILCIFTEATPHHPEFILTLVNNCPFTVWPAIQP
NSGHAVLERGGFALNTLTHHSFPAPAQHWSGRIWARTGCSYNHGHFSCATGDCG
GRLECNGLGGSSPATLAQFSLHHGHNDLSSYAVSLVDGFNVPMTVTPHEGKGLCP
VVGCRASLLATCPDRLQVRSPPGHGPVVACKSGCEAFGTDELCCRNHYNSPQTCR
ASSFSEFFKHACPATFTYAHDSPSLMHECSSPRELKIIFCH

>QsOLP XP_023907784.1 Quercus suber
MASSSFVFLTATFLILCTLTRATNPGLFLTLVNNCPFTVWPAIQPNSGHPVLERGGF
ALHTLTHHSFPAPTQHWSGRIWARTGCTYANNRFSCATGDCGNRLECDGLGGATP
ATLAQFSLHHGHTDLSSYAVSLVDGFNVPMTVTPHEGKGQCPVVGCRANLLATCP
DKLQLRSPPGHGPVVACKSGCEAFGTDELCCRNHYNSPQTCRASTFSEFFKHACPA
TFTYAHDSPSLMHECSSPRELKVIFCH

>GhOLP XP_016689054.1 Gossypium hirsutum
MASSLLVLATIFVLCSFGKASHPGLILTVVNNCPFTIYPAIQPNAGHPVLERGGFALH
TLTHRSFPAPTVHWSGRIWARTGCTYSNGHFSCATGDCGHRIECNGLGGATPVTIA
QFSLHHGGHKDLSSYEVSLVDGFNIPMTVTPHEGKGLCPVVGCRANILATCPGKLQ
YRSPPHGPVVGCKSGCAAFGTDELCCRNHYNSPQTCRASSYSEFFKHACPATFTYA
HDSPSLMHDCSSPRELKVIFCH

>GrOLP XP_012446484.1 Gossypium raimondii
MASSLLVLATIFVLCSFGKATHPGLILTVVNNCPFTIYPAIQPNAGHPVLERGGFAL
HTLTHRSFPAPTVHWSGRIWARTGCTYSNGHFSCATGDCGHRIECNGLGGATPVTI
AQFSLHHGGHKDLSSYEVSLVDGFNIPMTVTPHEGKGLCPVVGCRANILATCPGKL
QYRSPPHGPVVGCKSGCAAFGTDELCCRNHYNSPQTCRASSYSEFFKHACPATFTY
AHDSPSLMHDCSSPRELKVIFCH

>GaOLP XP_017650093.1 Gossypium arboreum
MASSLLVLATIFVLCSFGKATHPGLILTVVNNCPFTIYPAIQPNAGHPVLERGGFAL
HTLTHRSFPAPTVHWSGRIWARTGCTYSNGHFSCATGDCGHRIECNGLGGATPITI
AQFSLHHGGHKDLSSYEVSLVDGFNIPMTVTPHEGKGLCPVVGCRANILATCPGKL
QYRSPPHGPVVGCKSGCAAFGTDELCCRNHYNSPQTCRASSYSEFFKHACPATFTY
AHDSPSLMHDCSSPRELKVIFCH

>JcOLP XP_012065012.1 Jatropha curcas
MATATVLFSTISTALFILFSFGQAAPLILTLVNNCPFTVYPAIQPNAGHPVLEKGGFP
LSTLTHRSFPVPNQHWTGRIWARTGCTHTNGKFHCATGDCNHQLECNGLGGAPPA
TLAQFSLHHGHNDFSSYGVSLVDGFNLPMTITPHEGKGVCPVVGCRANLLTTCPQK
LQVRYPVGHGPVVACKSGCLAFGTDELCCRNHYNNPQTCRASSYSEFFKHACPAT
FTYAHDSPSLMHECSSPRELKVIFCH

>EsOLP XP_006409911.1 Eutrema salsugineum

MAKTSLPLAASFLLLLISSAAAADTGRLFLTVVNHCPFTVWPAIQPNAGHPVLEKG
GFALPTNTHRSFSAPATHWSGRIWGRTGCSHYNGKFSCVTGDCGHRLECNGLGGA
TPASLAQFDLHHGAHLDLSSYGVSLVDGFNVPMTVTPHEGRGVCPVVGCREDLLK
TCPAHLQLRSGHGGHVVACKSGCEAFRTDELCCRNHYNSPQTCRASSHSLFFKHA
CPSTMTFAHDSPSLMHDCSSPRELKVIFCH

>RsOLP XP_018463081.1 Raphanus sativus
MASRASLPLAASFLLLLISSSFAADTGRLFLTVVNHCPFTVWPAIQPNAGHPVLEKG
GFALPTNTHRSFSAPTSHWSGRIWGRTGCSHYNGKFSCVTGDCGNRLECNGLGGA
TPASLAQFDLHHGGHQDLSSYGVSLVDGFNVPMTVTPHEGRGVCPVVGCREDLLK
TCPAHLQLRSGHGGHVVACKSGCEAFRTDELCCRNHYNSPQTCRASTHSLFFKHA
CPSTMTFAHDSPSLMHDCSSPRELKVIFCH

>BnOLP XP_013676421.1 Brassica napus
MAKTSLPLAASFLLLLISSATSADTGRLFLTVVNHCPFTVWPAIQPNAGHPVLEKGG
FALPTNTHRSFLPPSTHWSGRIWGRTGCSHYNGKFSCVTGDCGHRLECNGLGGATP
ASLAQFDLHHGGHQDLSSYGVSLVDGFNVPMTVTPHEGRGVCPVVGCREDLLKT
CPAHLQLRSHGGHVVACKSGCEAFRTDELCCRNHYNSPQTCRASSHSLFFKHACPS
TMTFAHDSPSLMHDCSSPRELKVIFCH

>BrOLP XP_009140942.1 Brassica rapa
MAKTSLPLAASFLLLLISSTTSADTGRLFLTVVNHCPFTVWPAIQPNAGHPVLEKGG
FALLTNTHRSFFPPSTHWSGRIWGRTGCSHYNGKFSCVTGDCGHRLECNGLGGATP
ASLAQFDLHHGGHQDLSSYGVSLVDGFNVPMTVTPHEGRGVCPVVGCREDLLKT
CPAHLQLRSHGGHVVACKSGCEAFRTDELCCRNHYNSPQTCRASSHSLFFKHACPS
TMTFAHDSLSLMHDCSSPRELKVIFCH

>BooOLP XP_013632142.1 Brassica oleracea var. oleracea
MVKTSLPLTGFFFLLLISSSFAADTGRLFLTVVNHCPFTVWPAIQPNAGHPVLEKGG
FALPTNTHRSFSPPTTHWSGRIWGRTGCSHYNGKFSCVTGDCGNRLECNGLGGATP
ASLAQFDLHHGGHRDLSSYGVSLVDGFNVPMTVTPHEGRGVCPVVGCREDLLRTC
PAHLQLRSGHGGHVVACKSGCEAFRTDELCCRNHYNSPQTCRASSHSLFFKHACPS
TMTFAHDSPSLMHDCSSPRELKVIFCH

>BoOLP AAO12209.1 Brassica oleracea

MAKTSLPLAASFLLLLISSAAAADAGRLFLTVVNHCPFTVWPAIQPNAGHPVLEKG
GFALPTNTHRSFFPPSTHWSGRIWGRTGCSHYNGKFSCVTGDCGHRLECNGLGGA
TPASLAQFDLHHGGHQDLSSYGVSLVDGFNVPMTVTPHEGRGVCPVVGCREDLLK
TCPAHLQLRSHGGHVVACKSGCEAFRTDELCCRNHYNSPQTCRASSHSLFFKHACP
STMTFAHDSPSLMHDCSSPRELKVIFCH

>RcOLP XP_024159050.1 Rosa chinensis
MAHSHSLSLSLLSATLLILCSLAHATYPGHILTVVNNCPFPIWPAIQPNSGSPVLEKG
GFALPSLTHRSFPAPTQTWSGRIWARTGCSHSHNHFSCLTGDCGGKLECNGAGGA
TPATLAQFVLHHGPNDLWSYGVSLVDGFNIPMTVTPHEGKGVCPVVGCKANLLAT
CPDRLQVRSPAGHGPVVACKSACEAFGTDELCCRNKYNSPHTCRASSFSQYFKQA
CPATFTYAHDSPTLMHQCSSQRELKVIFCH

>OeOLP XP_022881590.1 Olea europaea
MAFPSTIFSLSLLLVLCTLPKATHAGLILTLVNNCPYTIWPAIQPNAGHPVLERGGFA
LHTLTHRSFPAPTTHWSGRVWARTGCTYAKGHFSCATGDCGGRLECEGLGGAAP
ATLAQFTLHHGHADFSSYGVSLVDGFNIPMTVTPHEGKGQCPVVGCRVDLLATCP
HGLKYYAPDGHVMGCKSGCTAFGTDELCCRNHYNSPQTCHASSYSAFFKHACPA
TVTYAHDNPSLMHECSSPRELKVIFCH

>FaOLP ABB86299.1 Ficus awkeotsang
MILTLVNNCPYPVWPAIQPNAGHPVLERGGFFLYSLTHRSFPAPTQHWSGRIWGRT
GCISAYNRFSCATGDCGGQLECNGAGGKTPATLVQLSLHHGHNDLSSYGVSLVDG
FNLGLTVTPHEGKGVCPVVGCRPDLLATCPDRLQVRSPHGHVVACKSGCDAFGTD
ELCCRNRYNSPQTCRASSYSAFFKHACPSSFTYAHDTPSLMHDCSSPRELKVIFCH

>CsOLP XP_010470049.1 Camelina sativa

MAKTSLPLAASFLLLISFSSAADSGRLFLTVVNNCPFTVWPAIQPNAGHPVLEKGGF
ALPTFTHRSFSAPTTHWSGRIWARTGCAHYNGKFSCVTGDCGNRLECNGLGGATP
ASLAQFDLHHGGHKDFSSYGVSLVDGYNVPMTVTPHEGHGVCPVVGCREDLLKT
CPGHLQVRSHAGHVVACKSGCEAFHSDELCCQGHYNSPNTCKASSHSLFFKHACP
STFTFAHDSPSLMHDCSSPRELKVIFCH

>ErgOLP XP_012827362.1 Erythranthe guttatus
MAATYLFPLSIFFIVCTVAKATHPGLILTVVNNCPYTIWPAIQPNAGHPALERGGFA
LSSLTHRSFPAPSAHWSGRLWARTGCIYAHGRFSCATGDCGGRLECGGLGGAAPA

TLAQFTLHHGHADFSSYSVSLVDGFNIPMTVTPHEGKGQCPVVGCRADLLATCPH
ALQVRGPPHGGVIGCKSGCAAFGTDELCCRNHYNSPQTCRASSYSEFFKHACPATF
TYAHDSPSLTHECSAPRELKVIFCH

>FvOLP XP_004294572.1 Fragaria vesca
MARSHSILLLSATLLILCSLAHATSPGHILTVVNNCPFPIWPAIQPNTGGQILERGGFF
LPSLSHRSFPAPTQSWSGRIWARTHCSRSNNRFSCLTGDCGGKLECNGAGGATPAT
LAQFVLHHGPNDLWSYGVSLVDGFNVPMTVTPHEGKGVCPVVGCKADLLATCPG
QLQVRSHPGVVACKSACEAFGSDELCCRNKYNSPQTCRASSFSQFFKHACPATFTY
AHDSPTLMHQCSSPRELKVIFCH

>InOLP XP_019199014.1 Ipomoea nil
MAAPISLFSLSLLLTLCVLCKATAPGLILTLVNNCPYTVWPAIQPNAGHPVLERGGF
ALNTLTHRSFAAPNAHWSGRIWARTGCTYAHGHFSCASGDCGGRLECDGAGGAT
PATLAQFVLHHGHADFSSYGVSLVDGFNLPMTVTPHEGKGKCPVVGCRANLLDTC
PAGLQFRSHGGHGPVVGCKSGCEAFKTDELCCRNHYNNPQTCKGSTYSEFFKHAC
PATFTYAHDSPSLMHECSAPRELKVIFCH

>NtOLP XM_018769102.1 Nicotiana tomentosiformis
MASSSTKTLLSLSLFLTLCALSQATYSGLILTLVNNCPYTIWPAIQPNAGHPVLERG
GFALHTLTHRSFAAPTTHWSGRIWARTGCSYAHGKFNCATGDCGGRIECDGLGGA
TPATLAQFVLHHGHADFSSYGVSLVDGFNIPLTVTPHEGKGQCPVVGCRAEILQTC
PAVLQLRSHGGHGPVVGCKSGCEAFKTDELCCRNHFNSPQTCKGSSYSEFFKHACP
ATFTYAHDSPSLMHECSSPRELKVIFCH

>NnOLP XP_010259948.1 Nelumbo nucifera

MTLLSSSASASRSSFLLFALSLVLLLLLCRSTLTHATSPGFILTVVNNCPFTIWPAIQPN
AGHVVLERGGFALQTLTHRSFPAPAHHWSGRLWARTGCTYSNGRFSCATGDCGGR
LECNGAGGVTPATLAQFSLHHGHNDQSSYGVSLVDGFNLPMTVTPHEGKGVCPVV
GCKANLLETCPKSLQLRCPPGHGPVVACKSGCEAFGTDELCCRNHYNSPQTCKASS
YAEFFKHACPATVTYAHDSPSLMHQCSSPRELKVIFCH

>ElgOLP XP_010918389.1 Elaeis guineensis

MASSSSSCSSFPLISLTFYSLLFLIVVQQVAAQYPAMTLTVVNNCPFTVWPAVQPNSG
HDVLEKGGFALSTLTHRSFPAPTHHWSGRIWGRTGCTFSGGRFSCATGDCGGRLEC
GGLGGAAPATLAQITLHHGGRRDLSSYGVSLVDGFNVGMTVTPHEGKGQCPVVGC

RANLLATCPDVLQVRAPVGGHVIGCKSGCQAFGTDELCCRNKYNNPRTCRASTYS
EFFKHACPATFTYAHDSPSLTHDCESPKELKVIFCH

>EgOLP XP_010033142.1 Eucalyptus grandis

MARAAASLLFPTAVLLLLLLAPSPSQALTLTLVNNCPFTVWPAIQPNAGHPVLERGG
FPLLTLTHRSFPHRRPWSGRVWARTACSHGHGGHFCATGDCGGRLECEGLGGATPA
TLAQFSLHHGHADFSSYAVSLVDGFNVPMTVTPHEGKGVCPVVGCRANLLATCPPH
LQMRSPAGHGPVVACKSGCEAFRTDELCCRNHFNSKDTCRPSSYSEFFKHACPATFT
YAHDSPSLMHECSSPRELKVIFCH

>NtOLP NP_001312698.1 Nicotiana tabacum

MASSSTKTLLSLSLFLTLCALSQATYSGLILTLVNNCPYTIWPAIQPNAGHPVLERGG
FALHTLTHRSFAAPTTHWSGRIWARTGCSYAHGKFNCATGDCGGRIECDGLGGATP
ATLAQFVLHHGHADFSSYGVSLVDGFNIPLTVTPHEGKGQCPVVGCRAEILQTCPAV
LQLRSHGGHGPVVGCKSGCEAFKTDELCCRNHFNSPQTCKGSSYSEFFKHACPATF
TYAHDSPSLMHECSSPHELKVILCH

>PaOLP XP_021820385.1 Prunus avium

MASSYHNNSLALLLTTFLILCSLTHATSPAPFLTLVNNCPFPLWPGIQPNSGHPVLER
GGFYLPALSHRSFPTPTQPWSGRIWARTHCTHTQNHFSCLTGDCGGRLECNGAGGA
APATLAQVSLHHGPNDLFSYGVSLVDGFNVPLTITPHEGHGVCPVVGCKADLLATC
PERLRVTSHAGVVACKSACEAFRSDELCCRNHYNSPQTCRASSYSEFFKRACPATFT
FAHDSPTLMHQCSSPHELKVIFCH

>AtOLP NP_001324474.1 Arabidopsis thaliana

MHIEKYFSLYTHTSHWHTFHTKSYTMAKTSLPLAASFLLLISFSSAVDTSRLFLTVV
NNCPFTVWPAIQPNAGHPVLEKGGFALPTFTHRSFNVPTTHWSGRIWARTWCAHY
NGKFSCLTGDCGNRLECNGLGGAPPASLAQFDLHHGGHHDFSSYGVSLVDGYNVP
MTVTPHEGHGVCPVVGCREDLIKTCPAHLQVRSHSGHVVACKSGCEAFHTDELCC
RGHYNSPNTCKASSHSLFFKHACPSSFTFAHDSPSLMHDCASPRELKVIFCH

>AlOLP XP_020885214.1 Arabidopsis lyrata

MHIKIYFSLYIYTHVALSHLSHQKLHTMAKTSLPLAASFLLLISFSSAVDTSHLFLTVV
NNCPFTVWPAIQPNAGHPVLEKGGFALPKYTHRSFNAPTTHWSGRIWARTGCAHY

NGKFSCITGDCGNRLECNGLGGAPPASLAQFDLHHGGHHDFSSYGVSLVDGYNVP
MTVTPHEGHGVCPVVGCREDLIKTCPAHLQVRSHSGHVVACKSGCEAFHTDELCC
RGHYNSPNTCKASSHSLFFKHACPSSFTFAHDSPSLMHDCSSPRELKVIFCH

>DcarOLP XP_017251860.1 *Daucus carota*
MAFFYHSLLIFISLSTSLSAATQQPFLLTIVNNCPFTIWPAIQPNYGHPVLENGGFCLP
ALSHRSFPAPTSPWSGRIWARTHCYTSPSHHFSCATGDCGTGKLECMGHGGSPPATL
AQFSLHHGPTDLSSYAVSLVDGFNVPMTVTPHEGHGVCPVVGCKGDLLATCPRAL
QVRASGGHVVGCKSACEAFGKDEFCCRGHFNSPQTCKPSSYSEFFKSACPSTFTYA
HDTPSLTHECSAPRELKVIFCH

>NsOLP XP_009800616.1 *Nicotiana sylvestris*
MASSSTKTLLSLSLCLTLCALSQATYSGLILTLVNNCPYTIWPAIQPNAGHPVLERGG
FALHTLTHRSFAAPTTHWSGRIWARTGCSYAHGKFSCATGDCGGRIECEGLGGATPA
TLAQFALHHGHADFSSYGVSLVDGFNIPLTVTPHEGKGQCPVVGCRAEILQTCPAVL
QLRSHGGHGPVVGCKSGCEAFKTDELCCRNHFNSPQTCKGSSYSEFFKHACPATFT
YAHDSPSLMHECSSPRELKIIFCH

>CpOLP XP_021887635.1 *Carica papaya*
MASSASSSSSSSLLLASTFLILFTLVKSTYPGLNITLVNNCPYTVWPAIQPNAGDPVLE
KGGFALHSLTHHSFAAPSQHWSGRFWGRTGCRYANGLFSCVTGDCGHRLECNGLG
GATPATLVEFSLHHGHNDFSSYRVSVVDGFNLPLTVTPHEGKGLCPVVGCRANLLE
TCPEKLQLRYGHGPVVACKSGCEAFKTDELCCRNHYNSPQTCRASSFSEFFKHACP
STFTYAHDSSSLLHECSSPRELKIIFCH

>GmOLP XP_003538000.1 *Glycine max*
MARSLFLCSLLIFAVTTFSSPTGALILTLVNNCNYTVWPAIQPNAGHPVLAGGGLTLR
TLTHQSIPVPDAHWSGRVWARTGCAYSGTAFSCASGDCGGRLQCNGAGGAPPATLA
QFEVHHGSNDYASYGVSLVDGFNVPMTVTPHEGKGVCPVVGCRDDLLATCPHVL
QHRVPAVHGPVVACKSGCEAFHTDELCCRNHFNNPSTCKGSIYSSFFKHAYPATFTF
AHDTPSLMHQCSSPRELKVIFCH

>CrOLP XP_006294865.1 *Capsella rubella*
MAQTSLPLAASFLLLISFSSAADTGRLFLTVVNNCPFTVWPAIQPNAGHPVLEKGGF
ALPTFTHRSFSAPTTHWSGRIWARTGCAHYNGKFSCITGDCGNRLECNGLGGATPA

SLAQFDLHHGGHKDFSSYGVSLVDGYNVPMTVTPHEGHGVCPVVGCREDLLKSCP
AHLQVRSHSGHVVACKSGCEAFRSDELCCRGHYNSPNTCKASSHSLFFKHACPSTF
TFAHDSPSLMHDCSSPRELKVIFCH

>NaOLP XP_019237711.1 Nicotiana attenuata
MASSSTKTLLSLSLFLTLCALSQATYSGLILTLVNNCPYTIWPAIQPNAGHPVLERGG
FALHTLTHRSFAAPTTHWSGRIWARTGCSYAHGKFNCATGDCGGRIECDGLGGAAP
ATLAQFALHHGHADFSSYGVSLVDGFNIPLTVTPHEGKGQCPVVGCRAEILQTCPAV
LQLRSHGGHGPVVGCKSGCEAFKTDELCCRNHFNSPQTCKGSSYSEFFKHACPATF
TYAHDSPSLMHECSSPRELKVIFCH

>MdOLP XP_008357503.1 Malus x domestica
MAVSRSHFHSLPLLSATTFLILAAVVHATHPQPSILTLVNNCPFPIWPAIQPNSGHPVL
ERGGFFLNALTHRSFPAPTQPWSGRIWARTHCTQNGPHLSCLTGDCGGRLECNGAG
GATPSTLAQISLHHGGPADLFSYGVSLVDGFNVPLTVTPHEGHGVCPVVGCKANLL
ATCPDRLKVTSPKGVVACKSACEAFGTDELCCRNHYNSPQTCRASGYSQFFKQACP
TTFTFAHDSPTLMHQCSSPRELKVIFCH

>DcatOLP XP_020683326.1 Dendrobium catenatum
MLPFFPLLSLLLISLPPPTFSQYPPSILTIVNNCPFTICPAIAPNSGHDVLEHGGFCLPTL
SHRSFPAPTHHWSGRIWGRTGCTHTGTHFSCATGDCGNRLECSGFTGATPATLAQLT
LHHGGHRDLVSYAVSLVDGFNLPMTVTPHEGRGLCPVVGCREDLLSTCPPELQVRF
PAGGSVVGCKSGCAAYGTDELCCRNMYNSPKTCRASTYSEFFKHHCPATFTYAHDS
PSLTHDCEGPKELKVIFCH

>SlOLP NP_001234714.1 Solanum lycopersicum
MASSSAKILLPLSLLFTLLSLSQSTNPNFILTLVNNCPYTIWPAIQPNAGHPVLERGGF
TLHSLTHRSFPAPNAHWSGRIWARTGCNYQHGKFYCATGDCGGRIECDGLGGAAP
ATLAQFVLHHGHADFSTYGVSLVDGFNIPLTVTPHEGKGVCPVVGCRANLLESCPA
VLQFRSHGGHGPVVGCKSACEAFKSDEFCCRNHYNSPQTCKPSSYSQFFKHACPAT
FTYAHDSPSLMHECSSPRELKVIFCH

Table S7. Anatomical characteristics of stem cross-section of the *PdOLP1* transgenics and wildtype plants.

**Poplar line**	**Phloem**		**Cambium**		**Xylem**		**Width ratio of xylem /phloem**
	**Number of cell layers**	**Width (μm)**	**Number of cell layers**	**Width (μm)**	**Number of cell layers**	**Width (μm)**	
Non-transgenic poplar	30-33	359.01	6–8	34.13	53-57	661.17	1.84
PdOLP1-OE OE3	28-31	311.81	5–7	29.89	43-48	527.99	1.69
PdOLP1-OE OE12	29-31	331.53	5–7	31.32	43-49	541.23	1.63
PdOLP1-OE OE21	29-32	339.68	6–7	32.74	44-49	552.28	1.63
PdOLP1-DR DR8	34-37	393.15	7–9	37.18	60-66	727.49	1.85
PdOLP1-DR DR15	34-38	402.05	7–10	39.04	60-67	763.45	1.90
PdOLP1-DR DR32	34-38	411.05	7–10	40.28	61-67	785.81	1.91

Notes :
PdOLP1-OE, *PdOLP1*-overexpressing lines; *PdOLP1*-DR, *PdOLP1*-downregulated lines. Cell layers, stems thicken by cell proliferation within the vascular cambium. The vascular cambium increases the number of cell layers in the phloem or xylem zones through periclinal division. The xylem and phloem regions show an orderly radial arrangement, so the cell layers can be accurately counted (see Figure 5a-f).

Table 8 Dry weight biomass, wood density and carbon storage of transgenic poplar and control plants

**Poplar line**	**Dry weight biomass (g)**	**Wood density unit: (g·cm^{-3})**	**Carbon storage (m^3)**
Non-transgenic poplar	46.13	0.76	0.137
PdOLP1-OE OE3	34.61	0.57	0.101
PdOLP1-OE OE12	35.89	0.62	0.103
PdOLP1-OE OE21	36.67	0.64	0.109

PdOLP1-DR DR8	57.54	0.89	0.174
PdOLP1-DR DR15	59.66	0.91	0.181
PdOLP1-DR DR32	62.77	0.94	0.186

Measurements were conducted on transgenic poplar plants overexpressing *PdOLP1* (OE3, OE12 and OE21), and *PdOLP1* downregulated plants (DR8, DR15 and DR32) and Non-transgenic poplar plant. The results are given as means ± SD of three independent assays. Asterisks indicate significant differences ($P < 0.05$) when compared with the control plants

Table S9. Binding sites in the promoter of the *PdOLP1* gene recognized by bZIP, WRKY, BLH, bHLH and MYB transcription factors.

The promoter of poplar *PdOLP1*

>*PdOLP1* promoter

ACGGCCCGGGCTGGTATCCTCTTTCGCCACCGTGTTTGTCGCCGACCACTGTTACCTCGATCTAA
AACACTCCTCTCTTTCTCTCAAGAATAACCCTCGGGGTTTTACACTTGAACTCCATGGACATGAA
TTTTGCTTCCCAATTAAACGAAGTGCATGAAAATATTGACCTGAATTAATTTTGTGGATAGACGA
CCAGCTAATTGAAAATATCGTTGAAAGAAACCATTTAAAATATCGTTTCCTTACTTAGTTTTCGT
GTGTGTGATTAGTGGTTTTTTAAAATATCGCACTACATTATAAACATTATTTTTTAAAAAAATAA
CACAGTTTGGTAAAAAAATTGAAAGAATAATATATATTGAAAAAATATTTAGAAAATATTATAA
TTTAAAAATAACTATGTTTTTTAATATTGTTTTTGTCCACCGCTGTTATATCATGGTTGTTTAGTTA
TATTTCAAGATGCATATTTTTTTAAAAAATAATTAACCTAAACCAAAGTTATCCCAAGAAAGATT
TTATGAAGATTTAATTGTCAAAAAATGGCTACTATTTAATTACTGTAAAAATAGCTACTATTTAA
TAACTGTAAATTACTGTCAAAAAATAGATTTAATGGTCAAGATTTTATCACTGTGTTAGAACTGT
AAAACACTCTTACATCGCAACACTCGCTAATTACATTTGTCCACTGCCACAATTGCGTACATGTA
ACACAGAACAACTGACAGCGCGGTGTCGTTAGGATGGAAAGAAGGCACTGGCGATGGGTCCCA
TCCCACGCTGCATATATACACGTATTACTTTTTCTCTTCTCACTTTGGCCTCTTGCCTTTCCTTTTT
GTTTTAAATTTGAACTGTGTTTGTGTTTATCGAATCAGACCTTCAGTACTGCTAAGATTGTGTTGG
GGAAGATGGTAGATGTCGAGGGTTGTGCATGGATCCACAGAAAAAAGCACGAAAAAACACGAA
AAGCTTATTTTTTATAGATAAGATTTTTATACATAGTTGAGTTGTACCTAACATCATCAATCTCGA
ACAGATGATGCTAGAACACTGATATGAGAAAACGAGGCCAATCCGAATCTACTTTATTAAAAAA
AAAATGCAAGCAAAAGTTAAAGTTTCACCACATTCCATGCTCTTTACAATGCTTCTTTATTAATT
ATTTAATTGTTTTGTTTTATTATACTCTTAACTTATCCATTTGCTAAATAACAGTTTGCAAATTAA
GGCACACAGGAAGCAAAATTTCTCAATGCTAAAATCAACTTACGGCCATTAACCAATATCAAAT
CTCTTCTTTCGCATTCAAAATTGACATTAGTTCTTGTAGGAAAAAATAAACATATGATTAGTTCT
ACGATTTTTTACATTTTTTTTCCTAAATGATTAAAATGCCACCACCTTCACGATGACGGTATAGGT
AGATGCACAAACTACAGAGTAATGGCGGCTTCTTCAAATGACCCCAAAGCCAGAAGAATCCCCC
TCCAATTTGCCCTTGGCATATTTCGGCTCCTGCATAATTCTATGACGATTCAATCCTTCTAACCAA
CTTTCAAACCAGAGGTATTCTAATTAATTGTAGTCGGCAGGGAAAGCTTTCTGAATTCCTGACGG
CTCAAGCCCTCAGAATTCAAAAGGACTAAAATACGTGCATCCTCATCTGAAAATACACAAAAGC
ACTCCAAAAAGCACATCTTCACCTTCCTCCCTCCCCACTCAACATTTAAGGAGAGGGGTATGTAT

GGTATTACGACATCACCCACAGAGGAAAAGAGAAAAGCTCGAAATCATGCATTCTTATCTCAAA
CCCTCGATGACTATAAGTAGTACACTTCCGGCACACTTGTCTCTTGAACCAAAAGGAAAAA

BLH, WRKY, bZIP, MYB, and bHLH binding sites in promoter of *PdOLP1*.

Gene name	BLH binding site	WRKY binding site	bZIP binding site	bHLH binding site	MYB binding site
PdOLP1	TGTCA (marked in red)	TGAC (marked in green)	ACACNNG (marked in pink)	CANNTG (marked in blue)	TATCC (marked in yellow)
